# Phosphoantigens glue butyrophilin 3A1 and 2A1 to activate Vγ9Vδ2 T cells

**DOI:** 10.1038/s41586-023-06525-3

**Published:** 2023-09-06

**Authors:** Linjie Yuan, Xianqiang Ma, Yunyun Yang, Yingying Qu, Xin Li, Xiaoyu Zhu, Weiwei Ma, Jianxin Duan, Jing Xue, Haoyu Yang, Jian-Wen Huang, Simin Yi, Mengting Zhang, Ningning Cai, Lin Zhang, Qingyang Ding, Kecheng Lai, Chang Liu, Lilan Zhang, Xinyi Liu, Yirong Yao, Shuqi Zhou, Xian Li, Panpan Shen, Qing Chang, Satish R. Malwal, Yuan He, Wenqi Li, Chunlai Chen, Chun-Chi Chen, Eric Oldfield, Rey-Ting Guo, Yonghui Zhang

**Affiliations:** 1 Tsinghua-Peking Center for Life Sciences, State Key Laboratory of Membrane Biology, School of Pharmaceutical Sciences, Tsinghua University, Beijing, China; 2 State Key Laboratory of Biocatalysis and Enzyme Engineering, Hubei Hongshan Laboratory, Hubei Collaborative Innovation Center for Green Transformation of Bio-Resources, Hubei Key Laboratory of Industrial Biotechnology, School of Life Sciences, Hubei University, Wuhan, China; 3 Department of Hematology, The First Affiliated Hospital of USTC, Division of Life Sciences and Medicine, University of Science and Technology of China, Hefei, China; 4 Schrödinger, Mannheim, Germany; 5 School of Medicine, Tsinghua University, Beijing, China; 6 School of Life Sciences, Tsinghua University, Beijing, China; 7 Beijing Advanced Innovation Center for Structural Biology, Technology Center for Protein Sciences, Tsinghua University, Beijing, China; 8 Department of Chemistry, University of Illinois at Urbana-Champaign, Urbana, IL USA; 9 Research Beyond Borders, Boehringer Ingelheim (China), Shanghai, China

**Keywords:** Immunology, Structural biology

## Abstract

In both cancer and infections, diseased cells are presented to human Vγ9Vδ2 T cells through an ‘inside out’ signalling process whereby structurally diverse phosphoantigen (pAg) molecules are sensed by the intracellular domain of butyrophilin BTN3A1^1–4^. Here we show how—in both humans and alpaca—multiple pAgs function as ‘molecular glues’ to promote heteromeric association between the intracellular domains of BTN3A1 and the structurally similar butyrophilin BTN2A1. X-ray crystallography studies visualized that engagement of BTN3A1 with pAgs forms a composite interface for direct binding to BTN2A1, with various pAg molecules each positioned at the centre of the interface and gluing the butyrophilins with distinct affinities. Our structural insights guided mutagenesis experiments that led to disruption of the intracellular BTN3A1–BTN2A1 association, abolishing pAg-mediated Vγ9Vδ2 T cell activation. Analyses using structure-based molecular-dynamics simulations, ^19^F-NMR investigations, chimeric receptor engineering and direct measurement of intercellular binding force revealed how pAg-mediated BTN2A1 association drives BTN3A1 intracellular fluctuations outwards in a thermodynamically favourable manner, thereby enabling BTN3A1 to push off from the BTN2A1 ectodomain to initiate T cell receptor–mediated γδ T cell activation. Practically, we harnessed the molecular-glue model for immunotherapeutics design, demonstrating chemical principles for developing both small-molecule activators and inhibitors of human γδ T cell function.

## Abstract (web summary)

Phosphoantigen-mediated BTN2A1 association drives BTN3A1 intracellular fluctuations outwards in a thermodynamically favourable manner, thereby enabling BTN3A1 to push off from the BTN2A1 ectodomain to initiate T cell receptor–mediated γδ T cell activation.

## Main

There are two families of T cells, αβ T cells and γδ T cells, defined by their T cell receptors (TCRs). Knowledge about how αβ T cells recognize their antigens has expanded greatly over the past 40 years^5^. The common feature of antigen recognition by αβ T cells is that all antigens bind in a groove formed by an extracellular region of major histocompatibility complex (MHC) (or MHC-like) antigen-presenting molecules, enabling direct interactions with αβ TCRs^5^. In contrast to αβ T cells, the molecular basis for antigen recognition by γδ T cells is not yet clear^6^. Vγ9Vδ2 T cells—a major subtype of human circulating γδ T cells—respond to multiple cancers and infectious diseases in an MHC-independent manner; they are specifically activated by small, non-peptidic diphosphate metabolites called pAgs. Studies in the 1990s showed that the universal precursors of isoprenoids, isopentenyl diphosphate (IPP) and dimethylallyl diphosphate (DMAPP)^7^ can weakly activate Vγ9Vδ2 T cells^8^, and these small molecules are known to accumulate intracellularly during tumorigenesis^9^. Later studies identified a hydroxy-analogue of DMAPP, (*E*)-1-hydroxy-2-methyl-but-2-enyl 4-diphosphate (HMBPP), as a very strong pAg of biological origin, with a half-maximum effective concentration (EC_50_) for Vγ9Vδ2 T cell activation in the picomolar range^10^. HMBPP is considered to be an exogenous pAg, and is produced by a range of pathogens through the methylerythritol 4-phosphate pathway^11^.

The sensing of pAgs in target cells requires a surface protein, BTN3A1^12–17^, that has both intracellular and extracellular domains. Although the pAg-binding site was initially linked to the extracellular domain^17^, this idea was later revised^1–3^, and there is now crystallography evidence that HMBPP binds to BTN3A1’s intracellular B30.2 domain^1,4^. This intracellular binding triggers an extracellular conformational change that allows target cells to be recognized by γδ T cells in a process that is known as inside-out signalling^1,4^. Our earlier studies indicated that a BTN3A1 monomer alone is unlikely to trigger the pAg-induced extracellular conformational change^4^. It has also been proposed that either BTN3A1 dimers^4,18^ or complexes with one (or more) accessory protein(s)^2,3,19–21^ are responsible for translating the intracellular pAg-recognition events to the extracellular changes to activate γδ T cells. Those findings have influenced our ongoing efforts to identify the accessory protein(s) that participate in conveying the signal from pAg sensing. During this period, BTN2A1 was shown to be a direct Vγ9 TCR ligand^22,23^, reconciling earlier reports showing that a BTNL3–BTNL8 heterodimer responds to the Vγ4 TCR^24^ and that the BTNL3 IgV domain directly interacts with the Vγ4 TCR^25^. Later, our group showed in a preprint that multiple pAgs can promote the association between the intracellular domains of BTN3A1 and BTN2A1^26^, and another study described what they termed the HMBPP receptor complex, comprising a bound pAg ligand and the intracellular domains of BTN2A1 and BTN3A1^27^. Thus, both BTN3A1 and BTN2A1 apparently exert dual functions as pAg sensors and as TCR ligands, although the structural basis for events inside and outside of cells has not been elucidated, and the discrete impacts of specific pAgs during γδ T cell activation remain unclear.

## Intracellular BTN2A1 does not bind to pAgs

**Fig. 1:**
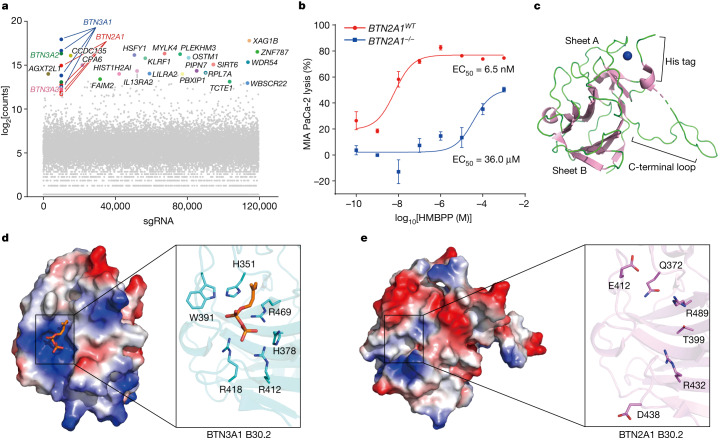
BTN2A1 is essential for pAg sensing but does not directly bind to pAgs. **a**, MIA PaCa-2 cells transfected with a whole-genome sgRNA library were pretreated with HMBPP (10 nM) for 4 h, and were then co-incubated with Vγ9Vδ2 T cells (sequentially, seven times) to enrich for genes related to target-cell killing. *BTN3A1* and *BTN2A1* (sgRNA ≥ 4) were identified in the screening. **b**, The cytotoxicity of Vγ9Vδ2 T cells towards *BTN2A1*^*WT*^ (red) or *BTN2A1*^*−/−*^ (blue) MIA PaCa-2 cells treated with HMBPP (100 pM to 1 mM). *n* = 4. Data are mean ± s.e.m. **c**, Cartoon model of the apo BTN2A1 B30.2 crystal structure (PDB: 8IGT). The two β-sheets, the extended C-terminal loop and the His tag are indicated. The zinc ion associated with the His tag is displayed as a blue sphere. **d**, Electrostatic surface of BTN3A1 B30.2 (left) and HMBPP-interacting residues (right). The highly cationic region (blue) and anionic regions (red) are shown. **e**, Electrostatic surface (left) and residues (right) in the BTN2A1 B30.2 structure corresponding to BTN3A1 B30.2 are illustrated. Source data

**Extended Data Fig. 1:**
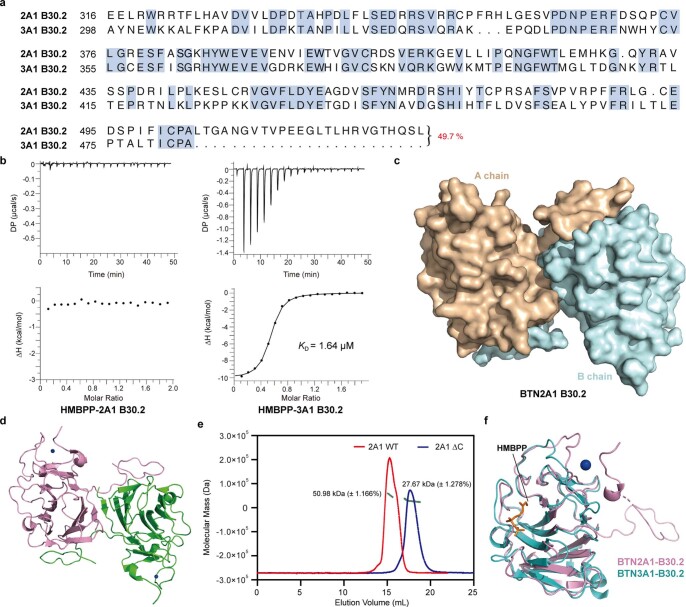
HMBPP binds to the BTN3A1 B30.2 domain but not to BTN2A1’s B30.2 domain. **a**, Sequence alignment of the B30.2 domains of 2A1 and 3A1 (49.7% similarity). **b**, ITC results for HMBPP binding to 2A1 B30.2 (left) and binding to 3A1 B30.2 (right). **c**, The dimer interface of 2A1 B30.2 buried approximately 1924 Å^2^, (PDBePISA server: https://www.ebi.ac.uk/pdbe/pisa/), which accounts for 16% of the total surface area of the monomer. **d**, The BTN2A1 B30.2 dimer. **e**, SEC-MALS analysis of 2A1 B30.2 and 2A1 B30.2-ΔC. 2A1 B30.2 (in red) exists mainly as a dimer in solution. The 2A1 B30.2-ΔC domain (in blue) exists mainly as a monomer in solution. **f**, Superimposition of 2A1 B30.2 (in pink) and HMBPP-bound 3A1 B30.2 (in cyan, PDB: 5ZXK). HMBPP is shown as a stick. Source data

Our study began with a genome-wide CRISPR–Cas9 knockout (KO) screen, which showed that the loss of four butyrophilins protected MIA PaCa-2 cells from γδ T cell killing: *BTN3A1*, *BTN3A2*, *BTN3A3* and *BTN2A1* (Fig. 1a). Notably, KO of *BTN2A1* increased the EC_50_ for HMBPP-mediated MIA PaCa-2 cell killing from 6.5 nM to 36.0 µM (Fig. 1b). BTN2A1 consists of extracellular domains (IgV and IgC), a transmembrane (TM) domain, a coiled-coil domain (JM), an intracellular B30.2 domain and a C-terminal tail. Although BTN2A1’s intracellular B30.2 domain shares around 50% sequence identity with BTN3A1’s intracellular B30.2 domain (Extended Data Fig. 1a), our isothermal titration calorimetry (ITC) assays revealed that the BTN2A1 B30.2 domain does not bind to HMBPP, in contrast to the strong interaction between HMBPP and the BTN3A1 B30.2 domain (Extended Data Fig. 1b).

We determined the crystal structure of BTN2A1 B30.2, which adopts the characteristic B30.2 fold comprising a β-sandwich formed by two sets of antiparallel β sheets (sheets A and B) for its SPRY domain (Fig. 1c and Extended Data Table 1; Protein Data Bank (PDB): 8IGT). The monomeric BTN2A1 B30.2 can form a homodimer with another molecule through crystallographic symmetry, with the dimer interface burying an area of approximately 1,924 Å^2^ (Extended Data Fig. 1c). The extended C-terminal loop in each monomer contributes to the homodimer interface (Extended Data Fig. 1d). Analysis using size-exclusion chromatography with multi-angle light scattering (SEC–MALS) confirmed that BTN2A1 B30.2 forms a homodimer in solution, and truncation of the C-terminal tail blocked homodimer formation (Extended Data Fig. 1e).

The structure of the BTN2A1 B30.2 domain closely resembles that of the BTN3A1 B30.2 domain (PDB: 5ZXK; Extended Data Fig. 1f). However, a notable difference is the presence of a basic pocket on the surface of the BTN3A1 B30.2 domain, which has previously been implicated in HMBPP binding^4^ (Fig. 1d). By contrast, no such basic pocket is evident in the corresponding region in the BTN2A1 B30.2 structure (Fig. 1e), explaining BTN2A1’s inability to bind to HMBPP. Note that the basic pocket and surrounding loops that bind to HMBPP in BTN3A1 are positioned in its intracellular B30.2/PRYSPRY region^4^, and similar regions in other B30.2-containing proteins have been implicated in protein–protein interactions^28,29^.

## HMBPP promotes BTN3A1–BTN2A1 association

**Fig. 2:**
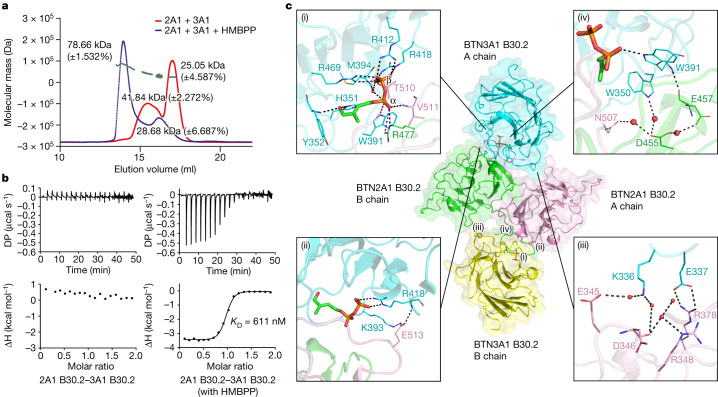
Exogenous HMBPP promotes BTN2A1 B30.2–BTN3A1 B30.2 association. **a**, SEC–MALS analysis of BTN2A1 B30.2–BTN3A1 B30.2 complexes with or without HMBPP. Numbers in plot indicate the molecular mass of each adjacent peak; green dashed line indicates control molecular mass. 2A1, BTN2A1; 3A1, BTN3A1. **b**, HMBPP promotes BTN2A1 B30.2 binding to BTN3A1 B30.2. **c**, The structure of the BTN3A1 B30.2–HMBPP–BTN2A1 B30.2 complex (PDB: 8JYE). Middle, cartoon representation of two BTN3A1 B30.2–HMBPP molecules (cyan and yellow) in complex with a BTN2A1 B30.2 homodimer (the A and B chains are shown in pink and green, respectively). Magnified views of the interactions between the BTN2A1 B30.2 dimer and the HMBPP–BTN3A1 B30.2 A chain are shown: HMBPP and the BTN2A1 and BTN3A1 B30.2 domains (i); BTN2A1 B30.2 and BTN3A1 B30.2 domains (ii–iii); and Trp350/Trp391 of BTN3A1 B30.2 and the BTN2A1 B30.2 domain (iv). Water molecules are shown as small red spheres. The black dashed lines indicate hydrogen bonds, and the purple dashed lines indicate salt bridges. Source data

**Extended Data Fig. 2:**
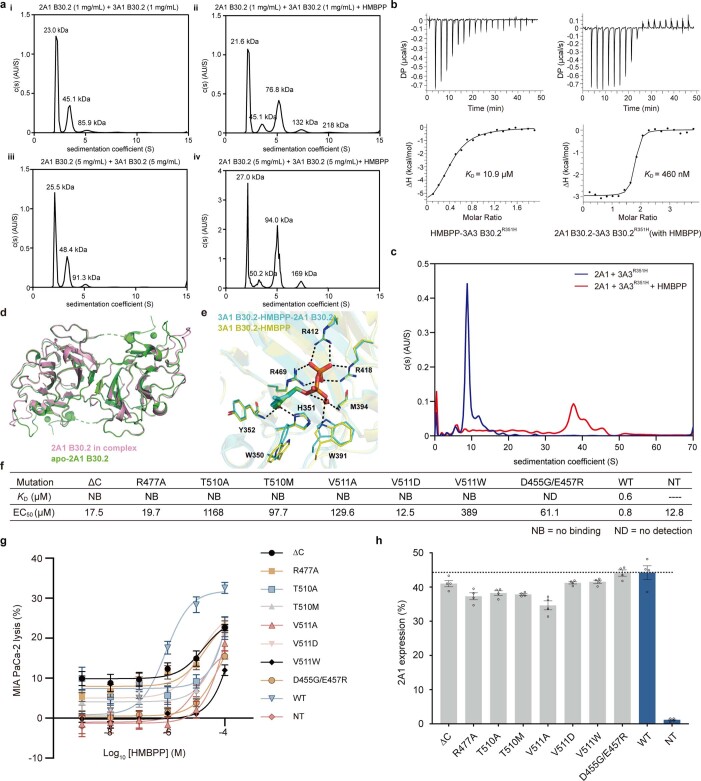
Features of BTN2A1 B30.2 required for its association with BTN3A1 and for HMBPP-induced Vγ9Vδ2 T cell activation. **a**, Sedimentation coefficient distribution c(s) profiles for different concentrations (1 mg/mL (i and ii) and 5 mg/mL (iii and iv) for each protein) of 2A1 B30.2 and 3A1 B30.2, with (ii and iv) or without (i and iii) HMBPP based on sedimentation velocity absorbance data. **b**, ITC results indicate that HMBPP binds to 3A3^R351H^ B30.2 (left) and promotes the 2A1 B30.2 binding to 3A3^R351H^ B30.2 (right). **c**, Sedimentation coefficient distribution c(s) profiles for 2A1 and 3A3^R351H^ at a 1:1.25 ratio with (red) or without (blue) HMBPP based on sedimentation velocity absorbance data. A new peak (37.6 S) was formed in the presence of HMBPP. **d**, Structure superimposition of apo-2A1 B30.2 and dimeric 2A1 in the 3A1 B30.2-HMBPP-2A1 B30.2 complex. **e**, An expanded view of HMBPP binding to 3A1 B30.2 (in yellow) (PDB: 5ZXK), showing the same interactions as observed in the 3A1 B30.2-HMBPP-2A1 B30.2 complex. **f**, ITC results for the indicated 2A1 B30.2 mutant variants binding to 3A1 B30.2 in the presence of HMBPP and the EC_50_ values of Vγ9Vδ2 T cells towards BTN2A^−/−^ MIA PaCa-2 (2A1/2A2 KO) transfected with plasmids for the indicated 2A1 mutant variants in the presence of HMBPP. **g**, Cytotoxicity of Vγ9Vδ2 T cells towards BTN2A^−/−^ MIA PaCa-2 (2A1/2A2 KO, *n* = 5, representative of two independent experiments) transfected with plasmids for the indicated 2A1 mutant variants, pretreated with HMBPP (1 nM~100 µM) overnight. Note that the 2A1 ΔC mutant was BTN2A1 with a truncated tail (truncated residues include LTGANGVTPEEGLTLHRVSLLE). Error bars: SEM. **h**, Flow cytometry analysis of His-tagged 2A1 mutant variants in BTN2A^−/−^ 293T cells (*n* = 4, representative of two independent experiments), as detected by anti-His mAb. Error bars: SEM. Source data

We next examined the interactions between BTN2A1 B30.2 and BTN3A1 B30.2. In the absence of HMBPP, no interaction was detected. However, the addition of HMBPP led to the formation of oligomers (a trimer, 78.7 kDa, as detected by a SEC–MALS (Fig. 2a); and a tetramer, 94.9 kDa, as detected by sedimentation velocity analytical ultracentrifugation (SV-AUC) (Extended Data Fig. 2a)). ITC confirmed that BTN2A1 B30.2 associates with BTN3A1 B30.2 in the presence of HMBPP (*K*_D_ = 611 nM) (Fig. 2b). Notably, introducing the R351H mutation into BTN3A3 (an inactive BTN3A isoform) enables it to detect HMBPP and to activate Vγ9Vδ2 T cells^1^. Our ITC studies indicated that HMBPP enhances the interaction between the B30.2 domain of this active mutant (BTN3A3(R351H)) and BTN2A1 B30.2 (*K*_D_ = 460 nM) (Extended Data Fig. 2b). Furthermore, SV-AUC analysis confirmed the specific association between these two proteins in the full-length form in the presence of HMBPP (Extended Data Fig. 2c). These results support that HMBPP functions as a molecular glue to drive γδ T cell activation by inducing association of the BTN3A1 and BTN2A1 B30.2 domains.

We next crystallized a complex of BTN3A1 B30.2, HMBPP and BTN2A1 B30.2 and, ultimately, solved the structure at a resolution of 2.18 Å (Fig. 2c and Extended Data Table 1; PDB: 8JYE). There are two BTN2A1 B30.2 molecules and two HMBPP-bound BTN3A1 B30.2 molecules in one asymmetric unit (Fig. 2c). The two BTN2A1 B30.2 polypeptides constitute a homodimer, basically as found in the apo-form BTN2A1 B30.2 structure (Extended Data Fig. 2d). BTN3A1 engagement with HMBPP forms a composite interface (Extended Data Fig. 2e) for direct binding to BTN2A1, with HMBPP positioned at the centre of the interface (Fig. 2c).

The Pα phosphate of HMBPP forms two interactions with BTN2A1 (Fig. 2c,i): one hydrogen bond and one salt bridge with Arg477 (BTN2A1, B chain), and one hydrogen bond with the main chain N atom of Val511 (BTN2A1, A chain, at 2.9 Å). The Pβ phosphate forms a hydrogen bond with Thr510 (BTN2A1, A chain, at 2.7 Å). To test whether the HMBPP-mediated BTN2A1–BTN3A1 association results in Vγ9Vδ2 T cell activation, we mutated BTN2A1’s Arg477, Thr510 and Val511 residues, each of which directly interacts with HMBPP. In contrast to wild-type (WT) BTN2A1, none of these BTN2A1 B30.2 mutants associated with BTN3A1 B30.2 or caused Vγ9Vδ2 T cell activation in the presence of HMBPP (Extended Data Fig. 2f–h), indicating that HMBPP’s ‘glue’ role is required for γδ T cell activation.

Several polar interactions were identified at the interfaces of BTN3A1 monomers and the BTN2A1 homodimer, including salt bridges and hydrogen bonds (Fig. 2c (ii and iii)). Notably, the main chain N atom of Trp391 (in BTN3A1) forms a hydrogen bond (at 2.9 Å) with the main chain O atom of Glu457 (BTN2A1, B chain), whereas Trp350 (BTN3A1) forms a water-mediated hydrogen bond (~3 Å) with Asp455 (BTN2A1, B chain) (Fig. 2c (iv)). Previous studies indicated that Trp350 and Trp391 of BTN3A1 B30.2 are key residues in γδ T cell activation^4,14^, but the precise nature of their contribution(s) remains unclear. We generated a BTN2A1 double mutant (D455G/E457R) to disrupt the interaction of those residues (Asp455 and Glu457) with BTN3A1’s Trp350 and Trp391 residues and found that these mutations significantly reduced the extent of HMBPP-triggered Vγ9Vδ2 T cell activation (Extended Data Fig. 2f–h).

## DMAPP and IPP are molecular glues

**Fig. 3:**
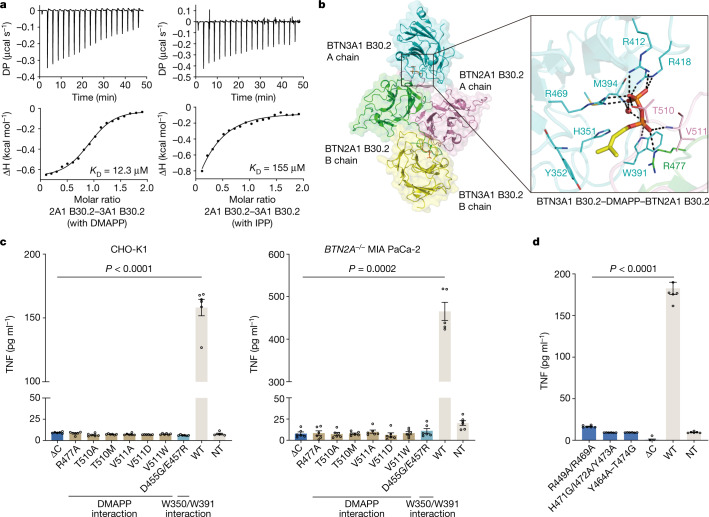
DMAPP and IPP function as molecule glues to activate γδ T cells. **a**, ITC analysis indicates that DMAPP and IPP promote the association between BTN3A1 B30.2 and BTN2A1 B30.2. **b**, Cartoon model of the BTN3A1 B30.2–DMAPP–BTN2A1 B30.2 complex (PDB: 8JYC). DMAPP is shown as a stick model and a water molecule is shown as a red sphere. **c**, TNF release by Vγ9Vδ2 T cells in response to zoledronate stimulation of BTN3A1^+^CD80^+^ CHO-K1 cells (left; *n* = 6) or *BTN2A*^*−/−*^ MIA PaCa-2 (*BTN2A1/BTN2A2* KO) cells (right; *n* = 5, representative of four independent experiments) transfected with the plasmids for the indicated BTN2A1 mutants. Residues in BTN2A1 B30.2 that directly interact with DMAPP and residues in BTN2A1 B30.2 that directly interact with Trp350/Trp391 in BTN3A1 B30.2 are indicated. Statistical analysis was performed using Welch’s analysis of variance (ANOVA) with Dunnett’s T3 multiple-comparison test, comparing each BTN2A1 mutant with the WT control. Data are mean ± s.e.m. **d**, TNF release by Vγ9Vδ2 T cells in response to zoledronate-treated (Zol, 10 µM) *BTN2A*^*−/−*^ MIA PaCa-2 cells (*n* = 6, representative of four independent experiments) that were transfected with plasmids encoding the indicated BTN2A1 mutants (based on the analysis in Extended Data Fig. 3d). Statistical analysis was performed using Welch’s ANOVA with Dunnett’s T3 multiple-comparison test, comparing with the WT control. Data are mean ± s.e.m. Source data

**Extended Data Fig. 3:**
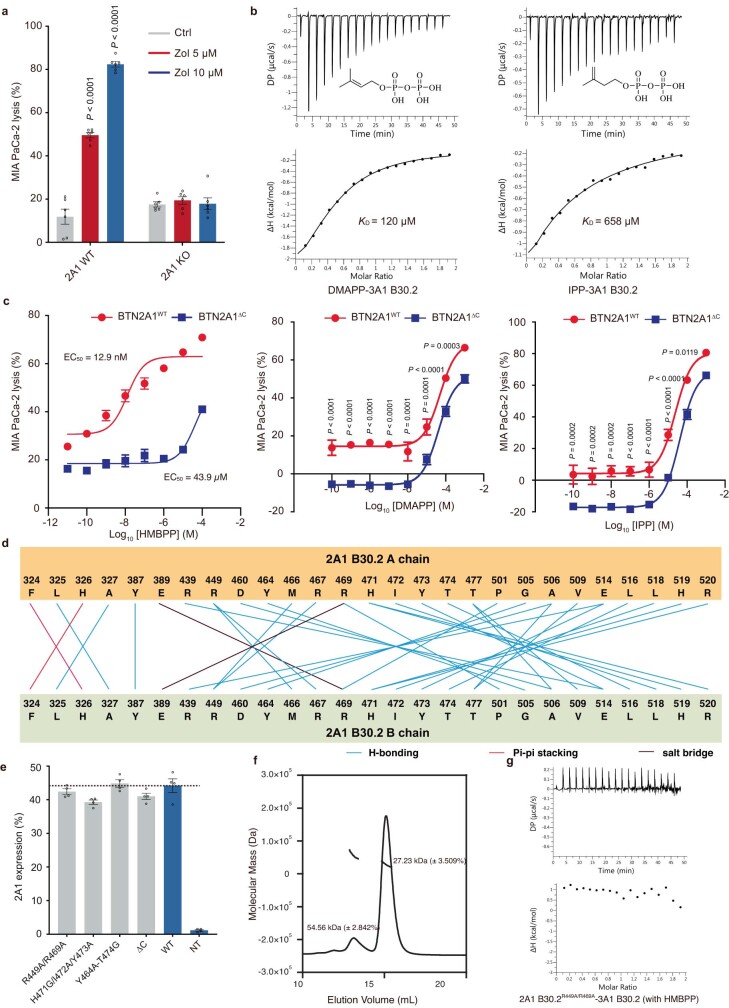
Disruption of the association of 2A1 B30.2 to 3A1 20.2 impairs γδ T cell responses to DMAPP and IPP. **a**, Cytotoxicity of Vγ9Vδ2 T cells towards BTN2A1^WT^ and BTN2A1^−/−^ MIA PaCa-2 cells (*n* = 6), which were pretreated with zoledronate (Zol) for 24 h. Data analysis: Two-way ANOVA with Šídák’s multiple comparisons test. *P* relative to the control. Error bars: SEM. **b**, ITC results for DMAPP and IPP binding to 3A1 B30.2. **c**, Cytotoxicity of Vγ9Vδ2 T cells towards BTN2A^−/−^ MIA PaCa-2 (2A1/2A2 KO) cells stably expressing 2A1 WT or ΔC mutant variants (*n* = 3), treated with different concentration HMBPP/DMAPP/IPP. Data analysis: Two-way ANOVA with Šídák’s multiple comparisons test. *P* indicated the comparison between two groups at equal concentrations. Error bars: SEM. Representative of three independent experiments in HMBPP/DMAPP group. Representative of two independent experiments in IPP group. **d**, Analysis of the interactions between 2A1 B30.2 A chain and B chain reveal residues affecting γδ T cell responses. **e**, Flow cytometry analysis of His-tagged 2A1 mutant variants in BTN2A^−/−^ 293T cells (*n* = 4, representative of two independent experiments), as detected by anti-His mAb. Error bars: SEM. **f**, SEC-MALS analysis of 2A1 B30.2^R449A/R469A^. The 2A1 B30.2^R449A/R469A^ exists mainly as a monomer in solution. **g**, ITC results indicate that 2A1 B30.2^R449A/R469A^ does not bind to 3A1 B30.2 in the presence of HMBPP. Source data

We next tested the ability of DMAPP and IPP, which are accumulated in tumorigenesis, to initiate the BTN3A1–BTN2A1 association. Zoledronate, a bisphosphonate drug, promotes γδ T cell activation by increasing DMAPP and IPP accumulation^30^. Consistent with a previous report^22^, we found that BTN2A1 is required for zoledronate-induced γδ T cell activation (Extended Data Fig. 3a). ITC analysis indicated that BTN3A1 B30.2 alone has a weaker binding affinity for DMAPP (120 μM) and IPP (658 μM) than for HMBPP (1.64 μM) (Extended Data Fig. 3b). However, both DMAPP and IPP promote the association between BTN3A1 and BTN2A1 B30.2, albeit to differing extents (*K*_D_ = 12.3 μM and 155 μM, respectively) (Fig. 3a). The binding affinity differences between DMAPP and IPP probably reflect the additional rotatable bonds in IPP and the resulting entropic penalties (as reflected in the computational analysis; Supplementary Fig. 2).

We determined the structure of the BTN3A1 B30.2–DMAPP–BTN2A1 B30.2 complex at a resolution of 2.29 Å (Fig. 3b and Extended Data Table 1; PDB: 8JYC). This complex closely resembles the BTN3A1 B30.2–HMBPP–BTN2A1 B30.2 complex (Figs. 2c and 3b and Supplementary Tables 1 and 2). However, in contrast to HMBPP, DMAPP does not form hydrogen bonds with the His351 and Tyr352 residues of BTN3A1 due to the absence of a 1-OH group (Fig. 3b).

Mutations in BTN2A1 B30.2 that disrupted Vγ9Vδ2 T cell activation by HMBPP also impaired activation by zoledronate (Fig. 3c). Truncation of the C-terminal tail of BTN2A1 reduced the killing of MIA PaCa-2 cells by Vγ9Vδ2 T cells induced by DMAPP and IPP (Extended Data Fig. 3c). Further mutations of BTN2A1 (mutation of the sequence encoding Tyr464–Thr474 to AAGAAGAAGAG, and mutation of additional residues as H471G/I472A/Y473A) designed to disrupt BTN2A1 dimerization (Extended Data Fig. 3d) significantly decreased γδ T cell responses to zoledronate (Fig. 3d and Extended Data Fig. 3e). Notably, a double mutant (BTN2A1(R449A/R469A)) reduced the extent of BTN2A1 dimer formation (Extended Data Fig. 3f), hindered the BTN3A1–BTN2A1 association (Extended Data Fig. 3g) and blocked zoledronate-mediated γδ T cell activation (Fig. 3d and Extended Data Fig. 3e). Collectively, these findings demonstrate that DMAPP and IPP act as molecular glues to drive γδ T cell activation.

## Conservation of the glue mechanism

**Fig. 4:**
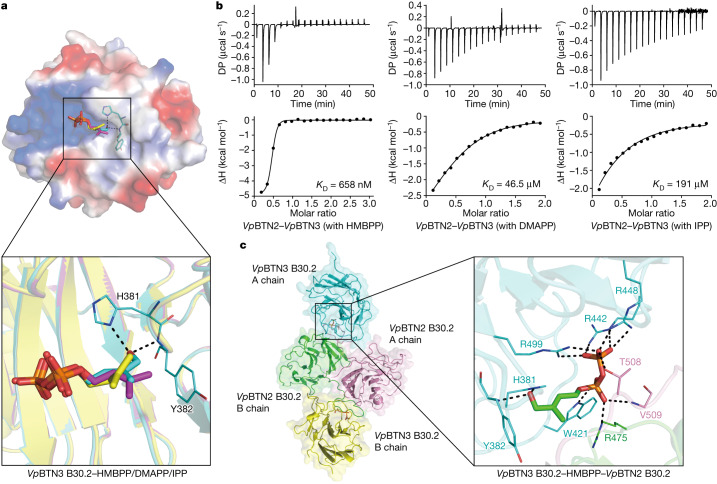
The molecular glue mechanism is conserved in alpaca. **a**, Structure superimposition of *Vp*BTN3 B30.2 (ΔC) in complex with HMBPP (cyan), DMAPP (purple) and IPP (yellow) (PDB: 8JY9, 8JYF and 8JYA, respectively). Electrostatic surface of HMBPP-bound *Vp*BTN3 B30.2 (up) and interacting residues of the HMBPP 1-OH group (down). **b**, ITC results for *Vp*BTN2 BFI binding to *Vp*BTN3 BFI in the presence of HMBPP, DMAPP and IPP. **c**, Cartoon model of the *Vp*BTN3 B30.2–HMBPP–*Vp*BTN2 B30.2 complex (PDB: 8HJT). HMBPP is shown as a stick model. The magnified view (right) illustrates the interaction networks formed by HMBPP and the three polypeptide chains.

**Extended Data Fig. 4:**
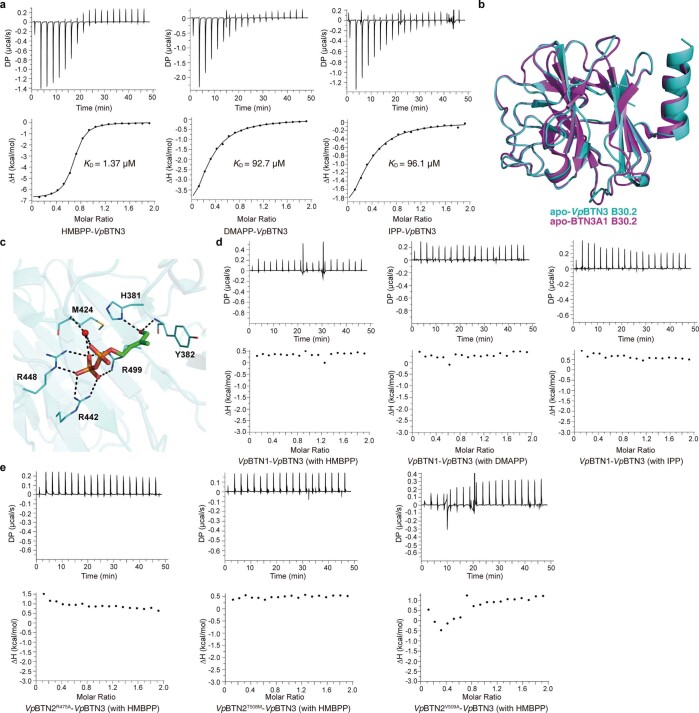
Structural and ITC studies reveals the key features involved in the association of *Vp*BTN3 and *Vp*BTN2 in the presence of pAgs. **a**, ITC results for HMBPP (left), DMAPP (middle), and IPP (right) binding to *Vp*BTN3 BFI. Notably, the *K*_*D*_ values of HMBPP and IPP have been reported previously^31^. **b**, Structure superimposition of apo-*Vp*BTN3 B30.2 (in cyan, PDB: 8JYB) and apo-BTN3A1 B30.2 (in purple, PDB: 4V1P). **c**, Expanded view of HMBPP and its interactions with protein residues in HMBPP-bound *Vp*BTN3 B30.2. HMBPP are shown as sticks and water is shown as small red spheres. **d**, ITC results indicate that *Vp*BTN1 does not bind to *Vp*BTN3 in the presence of HMBPP (left), DMAPP (middle) and IPP (right). **e**, ITC results indicate that mutations of the indicated conserved residues in *Vp*BTN2 (R475A, T508M and V509A, as revealed in Fig. 4c) abolished its binding to *Vp*BTN3 in the presence of HMBPP. Note that the residues in *Vp*BTN2 (R475, T508, and V509) correspond to 2A1 B30.2 residues (R477, T510 and V511) identified as functionally relevant for intracellular association with 3A1.

We observed a similar molecular glue behaviour of pAgs in alpacas (*Vicugna pacos*, *Vp*), to our knowledge the first non-primate species known to have a pAg-reactive Vγ9Vδ2 T cell subset^31^. Alpacas possess three butyrophilin molecules: *Vp*BTN3, *Vp*BTN2 and *Vp*BTN1. Among these, *Vp*BTN3 accommodates pAgs and is required for γδ T cell activation^31^. We found that HMBPP, DMAPP and IPP bind to *Vp*BTN3’s intracellular domain with varying affinities (*K*_D_ = 1.37 µM, 92.7 µM and 96.1 µM, respectively) (Extended Data Fig. 4a). The crystal structures of apo *Vp*BTN3 B30.2 (Extended Data Fig. 4b; PDB: 8JYB) or in complex with HMBPP, DMAPP and IPP (Fig. 4a, Extended Data Fig. 4c and Extended Data Table 2; PDB: 8JY9, 8JYF and 8JYA) showed that *Vp*BTN3 B30.2 can accommodate the pyrophosphate moiety of pAgs in a basic pocket (Fig. 4a).

HMBPP, DMAPP and IPP (in order of activity) promote the association between the *Vp*BTN3 butyrophilin full intracellular domain (BFI) and *Vp*BTN2 BFI (Fig. 4b), but not with the *Vp*BTN1 BFI (Extended Data Fig. 4d). A structural analysis revealed that *Vp*BTN2, resembling human BTN2A1, shares conserved residues (such as Arg475, Thr508 and Val509) that are essential for pAg-induced association with BTN3A1 (Supplementary Fig. 3). A *Vp*BTN2 BFI variant with mutations in these key residues abolishes its binding affinity for *Vp*BTN3 BFI in the presence of HMBPP (Extended Data Fig. 4e). The crystal structure of the *Vp*BTN3–HMBPP–*Vp*BTN2 complex (Fig. 4c and Extended Data Table 2; PDB: 8HJT) demonstrates similar binding behaviour between HMBPP and butyrophilins in humans and alpacas (Figs. 2c and 4c). Thus, in both humans and alpacas, structurally diverse pAgs function as molecular glues, enabling the formation of a composite interface between butyrophilins.

## Applications of molecular glue model

**Fig. 5:**
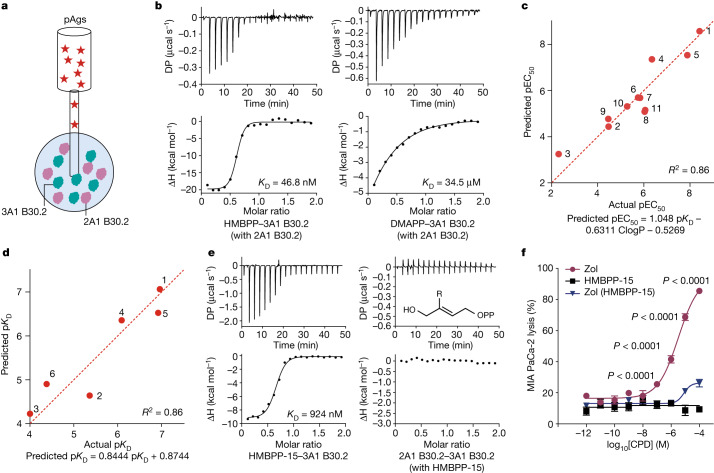
The molecular-glue model predicts pAg cellular activity and informs drug discovery. **a**, Schematic of ITC used to measure pAg binding affinity for the preconditioned BTN2A1–BTN3A1 complex. **b**, ITC results for HMBPP (left) and DMAPP (right) binding to the preconditioned BTN2A1–BTN3A1 B30.2 complex. **c**, The correlation between experimental and predicted pEC_50_ values (pEC_50_ = −log_10_[EC_50_ (M)]) for pAg-mediated Vγ9Vδ2 T cell killing of MIA PaCa-2 cells, obtained using the experimentally determined *K*_D_ value(s) (BTN2A1–BTN3A1 assay) and computed ClogP values (Supplementary Table 4) for a library of HMBPP analogues (structures are shown in Extended Data Fig. 5a). **d**, The correlation between experimental and predicted p*K*_D_ values (p*K*_D_ = −log_10_[*K*_D_ (M)]) for POP analogue binding to the preconditioned BTN3A1–BTN2A1 complex, obtained using the ITC experimentally determined *K*_D_ values (BTN2A1–BTN3A1 assay) and p*K*_D_ values computed by FEP+. **e**, ITC results demonstrating the binding of HMBPP-15 to BTN3A1 B30.2 (left) and ITC results showing that HMBPP-15 does not promote the interaction of BTN2A1 B30.2 with BTN3A1 B30.2 (right). OPP, diphosphate; R, 4-biphenyl. **f**, Cytotoxicity of Vγ9Vδ2 T cells towards MIA PaCa-2 cells stably expressing WT BTN2A1 that were treated with the indicated concentrations of HMBPP-15 or zoledronate (with or without HBMPP-15). CPD, compound. *n* = 6, representative of three independent experiments. Statistical analysis was performed using two-way ANOVA with Dunnett’s multiple-comparison test, relative to the WT control at equal concentrations. Data are mean ± s.e.m. Source data

The molecular glue model is applicable for investigating the cellular activities of diverse pAgs. A long-standing puzzle is that the cellular activities of pAgs, which can be as low as pM levels, do not correlate with their binding affinities to BTN3A1 B30.2, which are in the micromolar range^14^. To address this, we incubated BTN2A1 B30.2 and BTN3A1 B30.2 proteins together and measured the binding affinity of HMBPP to this preconditioned complex (Fig. 5a). Notably, we discovered a significantly enhanced binding affinity of HMBPP (*K*_D_ = 46.8 nM; Fig. 5b) compared with its affinity for BTN3A1 alone (*K*_D_ = 1.64 µM; Extended Data Fig. 1b), a value much closer to the observed cellular activity of HMBPP in γδ T cell activation. This enhancement can be attributed to multiple interactions between HMBPP and both BTN3A1 and BTN2A1 (Fig. 2c,i). For DMAPP, a far weaker γδ T cell activator than HMBPP, the *K*_D_ values went from 120 μM for BTN3A1 alone to 34.5 μM for the BTN2A1–BTN3A1 B30.2 complex (Fig. 5b and Extended Data Fig. 3b).

**Extended Data Fig. 5:**
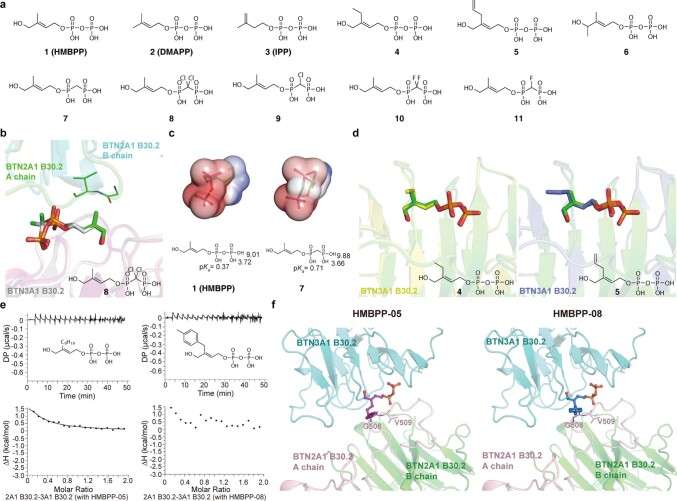
The molecular glue model explains the highly divergent cellular activities of specific pAgs. **a**, The chemical structures of diverse pAgs used in computational modelling. Compounds **4–6** are analogues of POP, while compounds **7–11** are analogues of PCP. Note that **7** has been reported^63^. **b**, The structure of 2A1-B30.2-HMBPP-3A1 B30.2 (PDB: 8JYE) was superimposed to the structure of 3A1 B30.2-compound **8** (PDB: 8IXV). **c**, Electrostatic potential surface of HMBPP (left) and its PCP analogue **7** (right), and the calculated p*K*_a_ values by Jaguar p*K*_a_ (Schrödinger Release 2023-1). **d**, The structure of 3A1 B30.2-compound **4** (PDB: 8IZE) and the structure of 3A1 B30.2-compound **5** (PDB: 8IZG) were superimposed to the structure of 3A1 B30.2-HMBPP (PDB: 5ZXK) respectively. **e**, ITC results indicate that HMBPP-05 (left) and HMBPP-08 (right) do not promote interaction of 2A1 B30.2 with 3A1 B30.2. **f**, The spatial hindrance posed by HMBPP analogues. The structures of 2A1 B30.2-HMBPP-3A1 B30.2 and HMBPP-08-bound 3A1 B30.2 (PDB: 6J06) were superimposed. It is clear that the bulky group of HMBPP-08 clashes with a loop at a 2A1 B30.2 region containing residues G508-V509. HMBPP-05 is docked into the BTN3A1 B30.2 based on the 2A1 B30.2-HMBPP-3A1 B30.2 complex, which clashes with the loop of 2A1 B30.2.

We further examined a series of HMBPP analogues that can be broadly classified as POP (diphosphate) or PCP (methylene diphosphate, as a POP isostere)^32^ analogues (all structures are shown in Extended Data Fig. 5a). Notably, we found a positive correlation (*R*^2^ = 0.86) was achieved between the experimentally observed Vγ9Vδ2 T cell killing activities (pEC_50_ = −log_10_[EC_50_ (M)]; Supplementary Table 4) and those predicted by using the experimental *K*_D_ and the computed ClogP values (Fig. 5c). Moreover, the BTN3A1–HMBPP–BTN2A1 structure enabled accurate prediction of the binding affinities of POP analogues using the computational Free Energy Perturbation (FEP+) program (Fig. 5d). The relatively weak activities of PCP analogues can also be explained by the molecular glue model, as their enhanced p*K*_a_ values lead to reduced binding to the basic residues of the butyrophilins (Extended Data Fig. 5b,c). By contrast, an analysis of crystal structures of HMBPP and its POP analogues (compounds **4** and **5**) in complex with BTN3A1 alone (PDB: 8IZE and 8IZG for compounds **4** and **5**, respectively; Extended Data Table 3) did not provide any insights with regard to their divergent activities; these structures showed almost identical binding modes (Extended Data Fig. 5d). These findings collectively illustrate the use of the molecular glue model as a conceptual framework for development of γδ T cell activators.

Disrupting the BTN3A1–BTN2A1 B30.2 association could potentially inhibit the activation of Vγ9Vδ2 T cells by natural pAgs, which would have implications for treatments of autoimmune diseases associated with γδ T cells. In a previous study^4^, we synthesized HMBPP-05 and HMBPP-08, two bulky HMBPP analogues that strongly bind to BTN3A1 B30.2 but do not efficiently activate γδ T cells. Here we found that both of these compounds (as well as HMBPP-15, a bulkier HMBPP-08 analogue that also binds tightly to BTN3A1; Fig. 5e) did not induce the BTN3A1–BTN2A1 B30.2 association (Fig. 5e and Extended Data Fig. 5e). A structural analysis immediately suggested that these bulky analogues would undergo steric clashes with the pocket formed by the BTN3A1 and BTN2A1 B30.2 domains (Extended Data Fig. 5f). Given their strong binding to BTN3A1 alone, and considering that the bulky substituents would preclude formation of the BTN3A1–BTN2A1 association, we anticipated that these compounds would inhibit activation of Vγ9Vδ2 T cells by natural pAgs. Indeed, this was confirmed when we pre-incubated MIA PaCa-2 cells with the representative bulky compound HMBPP-15, and observed blockage of zoledronate-sensitized killing by Vγ9Vδ2 T cells (Fig. 5f). Thus, our structural data and molecular glue insights can inform the rational design of immune therapeutics, including both activators and inhibitors of human γδ T cell function.

## Propagation of the pAg signal outwards

**Fig. 6:**
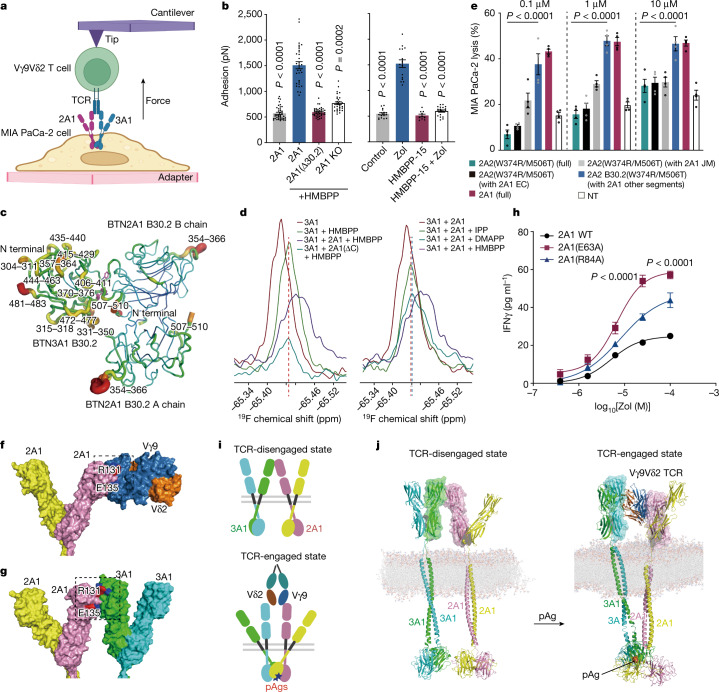
BTN3A1–BTN2A1 intracellular association leads to their extracellular push-off. **a**, Schematic of AFM-SCFS force measurement between a γδ T cell and a target cell. The schematic was created using BioRender. **b**, Disruption of the intracellular BTN3A1–BTN2A1 association reduced the pAg-enhanced adhesion force. Statistical analysis was performed using Kruskal–Wallis tests with Dunn’s multiple-comparison test, relative to the BTN2A1 HMBPP or zoledronate group. *n* = 40, 31, 39 and 31 (left) and *n* = 17, 16, 10 and 19 (right). Data are mean ± s.e.m. **c**, Molecular dynamics simulation showing that apo-structure regions (with root-mean-squared fluctuation (r.m.s.f.) > 1.5 Å) in the molecular dynamics trajectory map well to the crystal structure (the BTN3A1 B30.2–HMBPP–BTN2A1 B30.2 complex). Regions with high r.m.s.f. values are presented in red with increased thickness. **d**, The ^19^F-NMR chemical shift of TET–Cys357 (in BTN3A1) after binding to HMBPP and BTN2A1 B30.2 or its ΔC mutant (left). Right, comparison of the activities of HMBPP, DMAPP and IPP. **e**, The cytotoxicity of Vγ9Vδ2 T cells towards *BTN2A*^*−/−*^ MIA PaCa-2 cells (*n* = 4) that were transfected with plasmids encoding chimeric variants of BTN2A2 B30.2(W374R/M506T) and exposed to increasing concentrations of HMBPP (0.1 µM, 1 µM and 10 µM; above plots). In key, 'other segments' indicates extracellular (EC) and JM domains. Statistical analysis was performed using two-way ANOVA with Dunnett’s multiple-comparison test, relative to the ‘BTN2A2 B30.2(W374R/M506T) (with BTN2A1 other segments)’ group. Data are mean ± s.e.m. **f**, PIPER study of the interactions between extracellular BTN2A1 and the Vγ9Vδ2 T cell TCR. **g**, PIPER study of the interactions between extracellular BTN2A1 and BTN3A1. The overlapping BTN2A1 regions in **f** and **g** are coloured red. **h**, IFNγ release from Vγ9Vδ2 T cells in response to zoledronate stimulation of *BTN2A*^*−/−*^ MIA PaCa-2 (*BTN2A1/BTN2A2* KO) cells expressing mutant BTN2A1 variants (E63A and R84A). *n* = 6, representative of two independent experiments. Statistical analysis was performed using two-way ANOVA with Dunnett’s multiple-comparison test, relative to the WT control at equal concentrations. Data are mean ± s.e.m. **i**, Diagram of pAgs initiating inside-out conformational changes in BTN2A1–BTN3A1. **j**, Computational approaches to visualize inside-out signalling, showing the TCR-disengaged state and the TCR-engaged state of full-length BTN3A1 and BTN2A1. Source data

We investigated how pAg sensing is transmitted outwards to trigger Vγ9Vδ2 T cell TCR signalling by analysing cell–cell interactions and monitoring conformational changes in the butyrophilins. Using atomic-force single-cell microscopy (AFM-SCFS), we measured the binding force between a Vγ9Vδ2 T cell and a MIA PaCa-2 cell (Fig. 6a). HMBPP treatment significantly increased the force (from 558 pN to 1,554 pN), whereas *BTN2A1* KO or truncation of its C terminus to disrupt the BTN3A1–BTN2A1 association nullified this increase (Fig. 6b). Assays with zoledronate stimulation confirmed that HMBPP-15, which prevents the BTN3A1–BTN2A1 association, also reduced the binding force to basal levels (Fig. 6b). These results demonstrate that pAg sensing through the BTN3A1–BTN2A1 association strengthens the interaction between target cells and γδ T cells.

Previous studies have suggested that pAg sensing results in a conformational change in intracellular BTN3A1 that propagates outwards through BTN3A1’s JM region^15,18,33,34^. We performed molecular dynamics simulations using complex structures of BTN3A1 B30.2–pAg–BTN2A1 B30.2 with or without HMBPP or DMAPP to examine motion transmission (Fig. 6c). The simulations indicated that the BTN2A1 B30.2 domains remained rigid regardless of the presence of a pAg, whereas BTN3A1 B30.2 exhibited increased flexibility without a pAg (Supplementary Fig. 4a). Specifically, we identified a set of BTN3A1 residues with relatively large fluctuations (Fig. 6c; full trajectories are shown in Supplementary Fig. 4a). Clusters of HMBPP-interacting residues in BTN3A1 appeared to propagate fluctuations towards BTN3A1 ‘hot spots’ positioned in proximity to the JM region (residues 444–463, 435–440 and 357–364). To experimentally capture the BTN3A1 fluctuations, we conducted a ^19^F nuclear magnetic resonance (NMR) study. We labelled Cys357, an exposed cysteine residue in a BTN3A1 hot spot near the JM region (Supplementary Fig. 4b), with the ^19^F probe 2,2,2-trifluoroethanethiol (TET). Monitoring the ^19^F NMR signal after the addition of BTN2A1 B30.2 and various pAgs revealed significant changes, including chemical shifts and line broadening, most prominently with HMBPP, followed by DMAPP and IPP (Fig. 6d). Samples lacking BTN2A1 B30.2 or a variant with a truncated C-terminal tail did not show any changes in the TET signal (Fig. 6d). These findings emphasize that BTN2A1 drives inside-out signalling in a thermodynamically favourable manner. That is, compared with BTN3A1 alone, the additional and multiple intracellular binding interactions provided by BTN2A1 accelerate the inside-out signal propagation in an enthalpy-driven manner, with BTN2A1’s rigidity reducing the entropy penalty at the same time.

**Extended Data Fig. 6:**
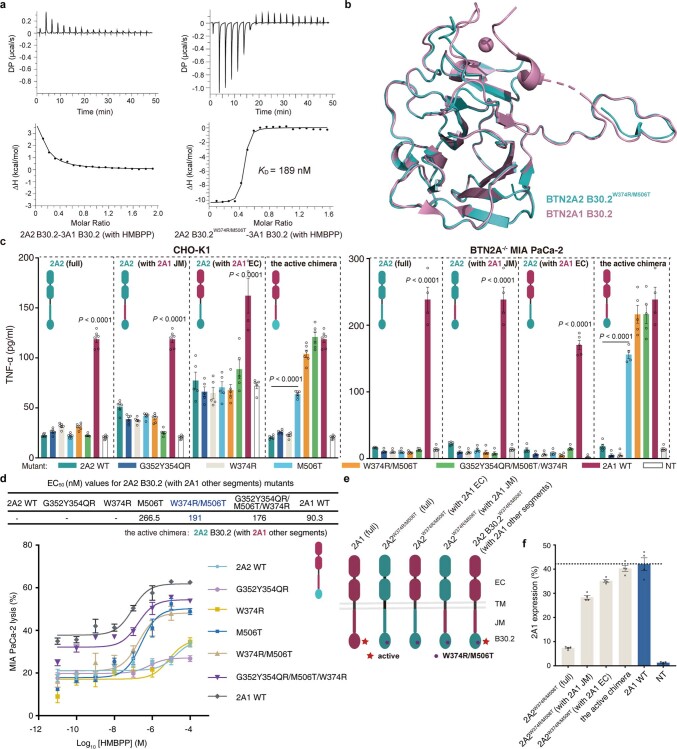
Engineering BTN2A2 for Vγ9Vδ2 T cell activation. **a**, 2A2 B30.2^W374R/M506T^ domain gains the function to bind to 3A1 30.2 in the presence of HMBPP. ITC results of the binding affinity of 2A2 (left) and 2A2 B30.2^W374R/M506T^ (right) to the 3A1 B30.2 domain in the presence of HMBPP. **b**, Structural superimposition of the engineered 2A2 B30.2^W374R/M506T^ domain (cyan, PDB: 8IH4) and the 2A1 B30.2 domain (pink, PDB: 8IGT). **c**, TNF-α release by Vγ9Vδ2 T cells in response to zoledronate (10 µM) stimulation of 3A1^+^CD80^+^ CHO-K1 cells (left, *n* = 5) or BTN2A^−/−^ MIA PaCa-2 cells (right, *n* = 5) transfected with plasmids encoding chimeric variants of 2A2 (with the 2A1 JM, EC, or both JM and EC, as indicated). Note that no “active” 2A2 chimera was obtained upon replacing the 2A2 JM or EC regions/segments with the counterpart sequences from 2A1, whereas the chimera comprising the 2A2 B30.2 domain with the 2A1 JM and EC segments was active for T cell activation (henceforth termed “the active chimera”). Data analysis: using Two-way ANOVA with Dunnett’s multiple comparisons test. *P* compared each 2A1 mutant to the WT control or 2A1^M506T^. Error bars: SEM. **d**, Top: Cytotoxicity of Vγ9Vδ2 T cells towards BTN2A^−/−^ MIA PaCa-2 (2A1/2A2 KO, *n* = 4) cells transfected with plasmids for the active chimera bearing the indicated mutations exposed to HMBPP (10 pM to 100 µM). Bottom: Dose-response curves for MIA PaCa-2 cell lysis by Vγ9Vδ2 T cells exposed to HMBPP. Error bars: SEM. **e**, Sketches for chimeric variants of 2A2 B30.2^W374R/M506T^ (with the 2A1 JM, extracellular domain (EC), or both JM and EC, as indicated). The red star indicates that Vγ9Vδ2 T cells are activated. **f**, Flow cytometry analysis of His tagged chimeric variants of 2A2 (with the 2A1 JM, EC, or both JM and EC, as indicated) in BTN2A^−/−^ 293T cells (*n* = 4, representative of two independent experiments), as detected by anti-His mAb. *n* = 4. Error bars: SEM. Source data

**Extended Data Fig. 7:**
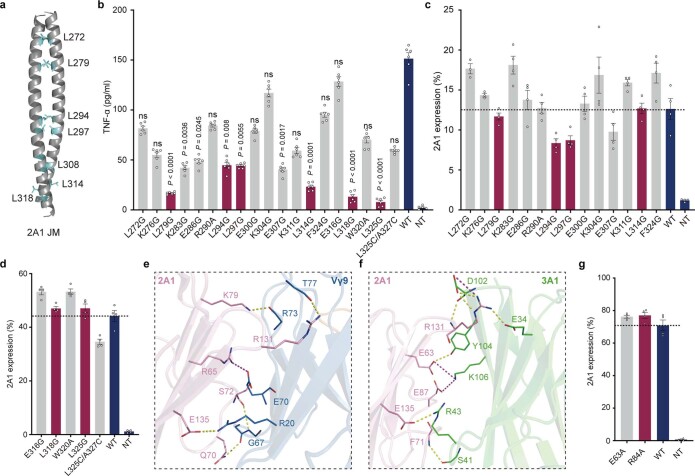
Computational and mutagenesis studies that characterize the role of 2A1 JM region and 2A1-Vγ9 (2A1-3A1) interaction. **a**, Structural predictions of the 2A1 JM region by the CCFold web server. Leucine residues are in cyan. **b**, TNF-α release by Vγ9Vδ2 T cells in response to zoledronate (Zol, 10 µM) stimulation of BTN2A^−/−^ MIA PaCa-2 cells (*n* = 6, representative of two independent experiments) transfected with the plasmids for the indicated 2A1 JM mutants. Data analysis: Kruskal–Wallis test with a Dunn’s multiple comparisons test. *P* relative to the WT control. Error bars: SEM. **c**, Flow cytometry analysis of His-tagged 2A1 JM mutant variants in BTN2A^−/−^ 293T cells (*n* = 4, representative of two independent experiments), as detected by anti-His mAb. Error bars: SEM. **d**, Flow cytometry analysis of His-tagged 2A1 JM mutant variants in BTN2A^−/−^ 293T cells (*n* = 4, representative of two independent experiments), as detected by anti-His mAb. Error bars: SEM. **e**, The predicted interactions between extracellular 2A1 and Vγ9. **f**, The predicted interactions between extracellular 2A1 and 3A1. **g**, Flow cytometry analysis of His-tagged 2A1 EC mutant variants in BTN2A^−/−^ MIA PaCa-2 cells (*n* = 4), as detected by anti-His mAb. Error bars: SEM. Source data

After introducing two gain-of-function mutations to the B30.2 domain of BTN2A2 (a BTN2A1 paralogue with 88.7% similarity), we observed efficient binding to BTN3A1 B30.2 in the presence of HMBPP (Extended Data Fig. 6a). Although the crystal structure of this variant closely resembled that of BTN2A1 B30.2 (Extended Data Fig. 6b; PDB: 8IH4), it did not activate Vγ9Vδ2 T cells (Extended Data Fig. 6c). Notably, Vγ9Vδ2 T cell activation occurred only when the JM domain, transmembrane domain and ectodomain of BTN2A2 were replaced with their BTN2A1 counterparts (Fig. 6e and Extended Data Fig. 6c–f). Computational predictions suggested that the JM region of BTN2A1 forms a coiled-coil dimer with a leucine-zipper-like heptad repeat (Extended Data Fig. 7a). To disrupt the propagation of the pAg sensing signal, we introduced specific mutations (L279G, L294G, L297G, L314G, L318G and L325G) that reduce the rigidity of BTN2A1’s JM domain, resulting in a significant reduction in the γδ T cell response to zoledronate (Extended Data Fig. 7b–d). Conversely, no reduction in the γδ T cell response was observed for the BTN2A1 (L325C/A327C) double mutant that we generated to firmly stabilize the homodimer (Extended Data Fig. 7b). These findings clarify that both a pAg and the specific presence of a (stable) BTN2A1 homodimer are required for the signal-propagating conformational change in BTN3A1.

## pAgs drive BTN3A1–BTN2A1 push-off outside

Previous studies have shown that the BTN2A1 ectodomain directly binds to the TCR Vγ9, promoting γδ T cell activation^22,23^. However, a ^1^H-^15^N NMR study revealed weak binding between the BTN2A1 ectodomain and the BTN3A1 ectodomain^23^, and recent studies have indicated that BTN2A1–TCR binding and BTN2A1–BTN3A1 ectodomain binding are mutually exclusive, owing to the close proximity and to the overlap of binding sites^35^. To investigate the potential extracellular consequences of the intracellular conformational changes in BTN3A1, we conducted computational studies using PIPER^36^ followed by molecular dynamics simulations to compare the TCR-engaged and TCR-disengaged states of the BTN2A1 and BTN3A1 ectodomains.

In the TCR-engaged state, PIPER predicted that Arg65 (BTN2A1) forms a salt bridge with Glu70 (TCR) and that Glu135 (BTN2A1) forms a hydrogen bond with Arg20 (TCR) (Fig. 6f and Extended Data Fig. 7e); note that all four of these residues have been implicated in the BTN2A1–TCR interaction^22,23^. In the TCR-disengaged state, the PIPER results indicated that the region of BTN2A1 that is involved in binding to BTN3A1 (including Arg131 and Glu135) overlaps with the known BTN2A1 reactive epitope^23^ for TCR interaction (Fig. 6g and Extended Data Fig. 7f). It is therefore plausible that the BTN2A1–BTN3A1 ectodomains need to be separated outside cells to expose BTN2A1’s reactive epitope for TCR engagement^35^. To test this, we individually mutated two BTN2A1 IgV residues (E63A and R84A) that are predicted to form salt-bridge interactions with BTN3A1 IgV but are not involved in interactions with the Vγ9 TCR. Assays with target cells expressing these BTN2A1 variants showed significantly enhanced γδ T cell activation after zoledronate stimulation (Fig. 6h and Extended Data Fig. 7g).

These findings support that the pAg-induced BTN3A1–BTN2A1 association inside cells enables BTN3A1 to ‘push off’ from the stable BTN2A1 homodimer, which can overcome its weak binding with the BTN2A1 ectodomain outside cells to facilitate TCR-mediated γδ T cell activation (Fig. 6i). A visualization of the whole inside-out signalling process, using computational approaches, is provided in Fig. 6j. It is also plausible that BTN3A1 and its known dimer partners BTN3A2 or BTN3A3^21,37^ can similarly push off from the stable BTN2A1 homodimer.

## Discussion

The previous proposal that direct loading of pAgs onto the BTN3A IgV ectodomain could form a complex interacting with the Vγ9Vδ2 TCR^17^, similar to the mechanism observed with MHC-type molecules in αβ T cells^38^, contradicts the fact that physiologically relevant pAgs are produced intracellularly^1^. Accordingly, focus shifted to BTN3A1’s intracellular domain, which was identified as the receptor for pAgs^10,14,15,17,20^. However, explaining the efficient immunosurveillance of Vγ9Vδ2 T cells has been challenging, owing to the disparity between the binding affinity of BTN3A1 (in the micromolar range) and the potent cell activity of the exogenous pAg HMBPP (in the picomolar range). Here we establish intracellular interactions between BTN2A1 and BTN3A1 as the basis for effective pAg sensing and inside-out signalling, ultimately triggering γδ T cell responses. The BTN3A1–pAg complex functions as a composite interface that directly binds to BTN2A1, bridged by the pAg molecule at the centre of the interface. The pAg therefore acts as a molecular glue to form the BTN3A1–BTN2A1 complex, enabling efficient immunosurveillance by Vγ9Vδ2 T cells. More speculatively, we posit that the evolution of this antigen-mediated, two-protein interaction represents a highly sensitive means for animals to detect otherwise difficult-to-perceive internal threats.

Our results indicate that pAg-mediated BTN2A1 association drives outward fluctuations of BTN3A1 within cells, enabling BTN3A1 to detach from the BTN2A1 ectodomain and initiate TCR-mediated γδ T cell activation. This mechanism deviates from αβ T cell activation and presents opportunities for therapeutic development. Targeting BTN3A and BTN2A1 holds promise as a therapeutic strategy for treating cancers and infectious diseases. Phase 1/2a clinical trials of a humanized anti-BTN3A monoclonal antibody (ICT01) have demonstrated efficacy against solid tumours and haematological malignancies^39^. Small-molecule drugs capable of mimicking pAgs as molecular glues offer an alternative to antibody drugs, providing convenient administration and potentially reduced costs. Our molecular glue model and structural data pave the way for designing activator and inhibitor molecules to modulate Vγ9Vδ2 T cell activation. In particular, our study emphasizes that developing activators based on BTN3A1 alone is unlikely to achieve the desired impacts, shifting the focus to targeting the BTN3A1–BTN2A1 association. Selectively blocking BTN3A1’s binding to BTN2A1 may enable selective inhibition of aberrant Vγ9Vδ2 T cell activation in autoimmune diseases. More broadly, considering the presence of B30.2 protein-binding domains in the intracellular tails of most butyrophilins^40^, which often require heteromeric interactions for immune function^21,24,25,41^, we anticipate that our finding that pAgs function as molecular glues linking BTN3A1–BTN2A1 inside target cells will contribute to investigations of the roles of other butyrophilins in immune modulation.

## Methods

### Cell lines

MIA PaCa-2 cells, a human pancreatic cancer cell line, were purchased from ATCC. HEK293T cells, a human embryonic kidney cell line, were purchased from ATCC. MIA PaCa-2 cells and HEK293T cells were cultured in Dulbeco’s modified Eagle’s medium (DMEM) (Gibco) supplemented with 10% heat-inactivated FBS and 1% penicillin–streptomycin (Beyotime) at 37 °C and 5% CO_2_. CHO-K1 cells, a subclone from the parental CHO cell line initiated from a biopsy of an ovary of an adult Chinese hamster, were purchased from ATCC. CHO-K1 cells were cultured in Ham’s F12K (Kaighn’s) medium (HyClone) supplemented with 10% heat-inactivated FBS, 1% penicillin–streptomycin at 37 °C and 5% CO_2_.

### Protein expression and purification

#### BTN3A1 B30.2

The BTN3A1 B30.2 domain was expressed and purified as described previously^1,4^.

#### BTN2A1 B30.2

The cDNA encoding the BTN2A1 B30.2 domain was cloned into the pET28a vector with an N-terminal or C-terminal 6×His tag. Overexpression of the BTN2A1 B30.2 domain was induced in *Escherichia coli* BL21 (DE3) cells by 1 mM isopropyl β-d-1-thiogalactopyranoside (IPTG) (Solarbio) at 18 °C for 24 h. Cells were collected by centrifuging, resuspended and lysed by sonication, and centrifuged at 20,000*g* for 1 h. The supernatant was collected and incubated with Ni-NTA resin (GE Healthcare), and was then eluted with a buffer containing 20 mM HEPES pH 7.5, 150 mM NaCl and 500 mM imidazole. The eluent was incubated overnight with TEV protease for 6×His tag cleavage, purified by Ni-NTA resin or further purified by DEAE Sepharose chromatography (GE Healthcare). Finally, the protein was dialysed against a storage buffer containing 20 mM HEPES pH 7.5 and 150 mM NaCl. The purified proteins were monitored at all stages of the purification process using SDS–PAGE and visualized by Coomassie blue staining.

All BTN2A1 B30.2-domain mutants were generated using a standard PCR mutagenesis strategy, overexpressed and purified in the same manner as for the WT protein.

#### BTN2A2 B30.2

The cDNA encoding the BTN2A2 B30.2 domain was also cloned into the pET28a vector with an N-terminal 6×His tag. The expression and purification of the BTN2A2 B30.2 domain and its mutants was performed according to the same methods as for the BTN2A1 B30.2 domain.

#### *Vp*BTN3 B30.2(ΔC) and *Vp*BTN1/2/3 BFI

The *Vp*BTN1/2/3 BFI (including *Vp*BTN2 BFI mutants) and *Vp*BTN3 B30.2(ΔC) domain were cloned, overexpressed and purified according to the same procedures as for the BTN2A1 B30.2 domain.

### Crystallization, data collection and structure determination

All crystals were obtained using the sitting-drop vapour-diffusion method. Final crystallization conditions were as follows: (1) apo BTN2A1 B30.2 domain (15 mg ml^−1^): 0.4 M potassium sodium tartrate; (2) BTN2A1–BTN3A1 B30.2 with HMBPP or DMAPP (25 mg ml^−1^ for each protein, molar ratio of BTN2A1:BTN3A1:HMBPP = 1:1.1:2 and BTN2A1:BTN3A1:DMAPP = 1:1.1:10, respectively): 42% v/v polyethylene glycol 200 and 0.1 M HEPES pH 7.5; (3) BTN2A2 B30.2^W374R/M506T^ domain (8 mg ml^−1^, 10 mM DTT): 0.2 M sodium citrate and 20% (w/v) polyethylene glycol 3350; (4) apo *Vp*BTN3 B30.2(ΔC) domain (8 mg ml^−1^, 10 mM DTT): 0.1 M Bis-Tris pH 5.5 and 2.0 M ammonium sulfate; (5) all of the complex crystals of *Vp*BTN3 B30.2(ΔC) were obtained by soaking HMBPP, DMAPP or IPP with apo *Vp*BTN3 B30.2(ΔC) crystals for about 72 h in mother liquor containing 0.1 M Bis-Tris pH 5.5 and 2.0 M ammonium sulfate; (6) *Vp*BTN2–*Vp*BTN3 B30.2 with HMBPP (10 mg ml^−1^ for each protein, molar ratio of *Vp*BTN2:*Vp*BTN3:HMBPP = 1:1:10): 0.1 M HEPES pH 7.5 and 12% (w/v) polyethylene glycol 8000; (7) the complex crystals of BTN3A1 B30.2 domain with compounds **4**, **5** and **8** were obtained based on our previously described procedures^4^.

The data of apo BTN2A1 B30.2 domain and BTN2A1–BTN3A1 B30.2 with HMBPP or DMAPP were collected at the Shanghai Synchrotron Radiation Facility (SSRF), beamlines BL17U1, BL18U1 and BL19U1, and data were processed using the HKL-2000 program.

X-ray diffraction data of BTN2A2 B30.2(W374R/M506T), apo *Vp*BTN3 B30.2(ΔC) and the complex crystals of *Vp*BTN3 B30.2(ΔC) with HMBPP, DMAPP and IPP, *Vp*BTN2–*Vp*BTN3 B30.2 with HMBPP and the BTN3A1 B30.2 domain with compound **8** were obtained at the in-house beamline (BRUKER D8 VENTURE) at Hubei University and datasets were processed with PROTEUM3 (Bruker AXS).

The data of the BTN3A1 B30.2 domain with compounds **4** and **5** were collected at beamline TPS05A of the National Synchrotron Radiation Research Center (NSRRC, Hsinchu, Taiwan) and processed using the HKL-2000 program.

Data collection and structure refinement statistics are shown in Extended Data Tables 1, 2 and 3. Final Ramachandran statistics were as follows: 97.5% favoured, 2.5% allowed and 0.0% outliers for the apo BTN2A1 B30.2 structure; 97.7% favoured, 2.3% allowed and 0.0% outliers for the BTN3A1 B30.2–HMBPP-BTN2A1 B30.2 structure; 96.3% favoured, 3.7% allowed and 0.0% outliers for the BTN3A1 B30.2–DMAPP–BTN2A1 B30.2 structure; 97.4% favoured, 2.6% allowed and 0.0% outliers for the BTN2A2 B30.2(W374R/M506T) structure; 98.7% favoured, 1.3% allowed and 0.0% outliers for the apo *Vp*BTN3 B30.2 ΔC structure; 98.1% favoured, 1.9% allowed and 0.0% outliers for the *Vp*BTN3 B30.2(ΔC)–HMBPP structure; 98.1% favoured, 1.9% allowed and 0.0% outliers for the *Vp*BTN3 B30.2(ΔC)–DMAPP structure; 98.7% favoured, 1.3% allowed and 0.0% outliers for the *Vp*BTN3 B30.2(ΔC)–IPP structure; 95.8% favoured, 4.2% allowed and 0.0% outliers for the *Vp*BTN3 B30.2–HMBPP–*Vp*BTN2 B30.2 structure; 98.9% favoured, 1.1% allowed and 0.0% outliers for the BTN3A1 B30.2-4–HMBPP structure; 98.9% favoured, 1.1% allowed and 0.0% outliers for the BTN3A1 B30.2-5–HMBPP structure; 97.8% favoured, 2.2% allowed and 0.0% outliers for the BTN3A1 B30.2-2Cl–HMBPP structure.

All structures were refined with COOT and REFMAC^42–44^. All protein structure figures were prepared using PyMOL (http://pymol.sourceforge.net).

### Vγ9Vδ2 T cell isolation and expansion

All studies using Vγ9Vδ2 T cells were performed in accordance with the recommendations of the Institutional Review Board of Tsinghua University with written informed consent from all of the participants. All of the participants gave written informed consent in accordance with the Declaration of Helsinki. The protocol was approved by the Institutional Review Board of Tsinghua University (project no. 20170004 and 20170007). Peripheral blood mononuclear cells were isolated from healthy donors by density-gradient centrifugation using Ficoll-Hypaque density fluid (GE Healthcare). Peripheral blood mononuclear cells were cultured at a density of 2 × 10^6^ cells per ml in RPMI 1640 (Gibco) that was supplemented with 10% FBS, 1% penicillin–streptomycin, 150 U ml^−1^ human rIL-2 (PeproTech), 1% MEM non-essential amino acids (Gibco), 2 mM l-glutamine (Beyotime), 50 mM β-mercaptoethanol (Amresco) and 5 µM zoledronate (Energy Chemical) at 37 °C and 5% CO_2_. Fresh medium containing human IL-2 was replaced every 3 days. Vγ9Vδ2 T cells (purity > 90%) were collected at 11 days and stored for future use.

### Viral library production

The whole-genome sgRNA library plasmid (GeCKOv2, a gift from D. Huang) plus packaging plasmids using FuGENE6 (Promega) were transfected into low-passage HEK293T cells at 80% confluency in 15 cm tissue culture dishes. Viral supernatants were collected at 48–72 h after transfection, filtered through a 0.45 µm filtration unit and stored at −80 °C for future use. Viruses for other plasmids (including Cas9, BTN2A1, BTN3A1, CD80 and luciferase) were produced in a similar manner.

### Whole-genome CRISPR–Cas9 KO screen

The CRISPR–Cas9 KO screen was performed essentially as described previously^45–47^. MIA PaCa-2 cells were infected with the lentiviral supernatant containing Cas9 at approximately 80% confluence in the presence of polybrene, and Cas9-positive MIA PaCa-2 cells were then sorted by fluorescence-activated cell sorting 48 h later. Sorted cells were infected with the lentiviral-packaged whole-genome sgRNA library to achieve 30% transduction efficiency, and transduced cells were selected with puromycin for an additional 10 days. MIA PaCa-2 cells (>100× library coverage) were pretreated with HMBPP (10 nM) for 4 h, and then co-incubated with Vγ9Vδ2 T cells at a T cell:cancer cell ratio of 10:1. After 72 h, Vγ9Vδ2 T cells were removed and remnant MIA PaCa-2 cells were re-expanded for 1–2 weeks. This process was repeated an additional ten consecutive times. Finally, genome sequencing was conducted for cells at each step, and sgRNA counting was performed and analysed as reported previously^47,48^. The raw count files and analysis script are provided as Source Data.

### Generation of *BTN2A1/BTN2A2-*KO cells

The *BTN2A1* and *BTN2A2* genes were disrupted in MIA PaCa-2 cells and HEK293T cells using CRISPR. The CRISPR sequencing targeting functional *BTN2A* genes was designed with the help of online tools (http://crispr.mit.edu) mentioned in Supplementary Table 5 and sequences were cloned into a PX458-pSpCas9(BB)-2A-GFP-MCS vector. All of the plasmids were sequenced to confirm successful ligation. The plasmids were then transfected into MIA PaCa-2 and HEK293T cells using Lipofectamine 2000 reagent. Single-cell clones were sorted (GFP) into 96-well plates using the FACSAria II (BD) system, and were then validated on the basis of genomic sequencing.

### Cell killing assays

MIA PaCa-2 cells were infected with lentivirus bearing luciferase transgene. Luciferase-positive cells were plated at 5.0 × 10^3^ cells per well in 384-well plates 1 day before transfection. All plasmids were transfected into *BTN2A*^*−/−*^ MIA PaCa-2 (*BTN2A1/BTN2A2* KO) cells using Lipofectamine 2000 reagent. After 48 h, MIA PaCa-2 cells, which were pretreated with different concentrations of phosphoantigen (HMBPP for 12 h, DMAPP or IPP for 4 h), were co-cultured with 1.0 × 10^4^ Vγ9Vδ2 T cells for 16 h. Stable-lite luciferase (Vazyme) was added to each well and the luciferase signal was immediately measured by using the PerkinElmer EnVision microplate reader.

Percentage of lysis was calculated using the following equation: percentage of specific lysis = (maximum luciferase − experimental luciferase)/maximum luciferase × 100.

### HMBPP-15-blocked zoledronate-sensitized killing by Vγ9Vδ2 T cells

MIA PaCa-2 cells were plated at 1.0 × 10^4^ cells per well in 96-well plates. After 4 h pretreatment with HMBPP-15 (50 µM), the plates were washed twice to remove HMBPP-15. MIA PaCa-2 cells were then treated with zoledronate (1 pM–100 µM) for 24 h. The medium was aspirated and cells were washed four times with PBS. Vγ9Vδ2 T cells (2.0 × 10^4^ cells per well) were then added and cocultured with MIA PaCa-2 cells. Stable-lite luciferase (Vazyme) was added to each well, and the luciferase signal was immediately measured using the PerkinElmer EnVision microplate reader.

### TNF secretion assays of Vγ9Vδ2 T cells co-cultured with *BTN2A*^*−/−*^ MIA PaCa-2 cells or CHO-K1 cells

*BTN2A*^*−/−*^ MIA PaCa-2 (*BTN2A1/BTN2A2* KO) cells were plated at 1.0 × 10^4^ cells per well in 96-well plates 1 day before transfection. All plasmids were transfected into MIA PaCa-2 cells using Lipofectamine 2000 reagent, followed by treatment with zoledronate (10 µM) for 24 h. The medium was then aspirated and cells were washed four times with PBS at room temperature. Vγ9Vδ2 T cells (1.0 × 10^5^ cells per well) were then added and cocultured with MIA PaCa-2 cells, and culture supernatants were collected after 16 h and assayed for TNF levels using a TNF human uncoated ELISA kit (Invitrogen). The assay of CHO-K1 cells (infected with lentivirus of BTN3A1 and CD80) was performed according to the same methods as for the MIA PaCa-2 cells^22,23^.

### Flow cytometry

To detect the BTN2A1 expression level at the plasma membrane, *BTN2A*^*−/−*^ HEK293T (*BTN2A1/BTN2A2* KO) cells were transfected with WT or mutant plasmids with an N-terminal 6×His tag, and cells were then stained with APC/PE anti-His antibodies (BioLegend) for 30 min at 4 °C. The percentage of BTN2A1 was measured using the LSRFortessa (BD) system.

To generate BTN2A1 stable expression cells, *BTN2A*^*−/−*^ MIA PaCa-2 (*BTN2A1/BTN2A2* KO) cells were infected with lentivirus bearing *BTN2A1* WT or ΔC-mutant transgene with an N-terminal 6×His tag. Cells were stained with APC/PE anti-His antibody for 30 min at 4 °C, and APC/PE-positive cells were then sorted using the Moflo Astrios EQ (Beckman Coulter) system. All data were analysed using FlowJo (BD).

### ITC

ITC experiments were performed using the MicroCal PEAQ-ITC instrument (GE Healthcare) at 25 °C. We used an initial injection of 0.4 μl followed by 19 injections (2 µl each) at 150 s time intervals. ITC binding fits were calculated using MicroCal Analysis software. The samples were prepared using a buffer (20 mM HEPES pH 7.5, 150 mM NaCl).

For pAg binding to the BTN3A1 B30.2 domain, the sample cell and injection syringe were filled with the buffer containing 100 µM protein and 1 mM HMBPP, respectively, or 400 µM protein and 4 mM DMAPP (IPP), respectively.

For HMBPP binding to the preconditioned BTN2A1 B30.2 and BTN3A1 B30.2 complex, BTN2A1 B30.2 (20 µM) and BTN3A1 B30.2 (10 µM) were incubated in the buffer at a 2:1 ratio and loaded into the sample cell, and the injection syringe was filled with buffer containing 100 µM HMBPP.

For DMAPP/IPP binding to the preconditioned BTN2A1 B30.2 and BTN3A1 B30.2 complex, BTN2A1 B30.2 (200 µM) and BTN3A1 B30.2 (100 µM) were incubated in buffer at a 2:1 ratio and loaded into the sample cell, and the injection syringe was filled with buffer containing 1 mM DMAPP/IPP.

For other pAgs, their concentrations were adjusted to 500 µM, and BTN2A1 B30.2 and BTN3A1 B30.2 were adjusted to 100 µM and 50 µM at a 2:1 ratio.

For the experiments testing the binding of BTN2A1/BTN2A2 B30.2 domain (including their mutants) to the BTN3A1 B30.2 domain with or without HMBPP, BTN3A1 B30.2 (100 µM) and HMBPP (300 µM) were incubated in the buffer at a 1:3 ratio and loaded into the sample cell, and the injection syringe was filled with the buffer containing 1 mM BTN2A1/BTN2A2 B30.2 (including their mutants).

For the BTN2A1 B30.2 domain binding to the BTN3A1 B30.2 domain in the presence of DMAPP or IPP, the concentration of BTN2A1 B30.2 was adjusted to 2 mM, and the concentrations of BTN3A1 B30.2 and DMAPP (or IPP) were adjusted to 200 µM and 2,000 µM at a 1:10 ratio.

ITC experiments of HMBPP, DMAPP or IPP binding to the *Vp*BTN3 BFI (or the preconditioned *Vp*BTN3 BFI and *Vp*BTN2 BFI complex) and *Vp*BTN2 BFI (or *Vp*BTN1 BFI) binding to *Vp*BTN3 BFI in the presence of HMBPP, DMAPP or IPP were performed according to the same procedures as for the BTN3A1 B30.2 and BTN2A1 B30.2.

### SEC–MALS

SEC–MALS experiments were performed using the Wyatt Dawn Heleos II multiangle light-scattering detector (Wyatt Technology) coupled to an AKTA Purifier UPC10 FPLC protein purification system and the Superdex 200 size-exclusion column (GE Healthcare). Protein molecular masses of the individual peaks observed in the size-exclusion chromatograms were analysed by static light scattering in conjunction with their corresponding refractive indices, using an online refractometer connected downstream of the static light scattering detector (Wyatt Optilab rEX). The proteins were prepared at 5 mg ml^−1^ in a buffer (20 mM HEPES pH 7.5, 150 mM NaCl). HMBPP was prepared in 0.5 mM. The experiments were performed with a running buffer (20 mM HEPES pH 7.5, 150 mM NaCl) at a flow rate of 0.5 ml min^−1^. A standard value of the refractive index, d*n*/d*c* = 0.185 ml g^−1^, was used for all proteins.

### AUC

Sedimentation experiments were performed at 20 °C and 45,000 rpm on the Beckman Coulter ProteomeLab XL-I analytical ultracentrifuge according to standard protocols^49^. All of the samples were prepared in the buffer (20 mM HEPES pH 7.5, 150 mM NaCl) and inserted into 12 mm (or 3 mm) Epon centerpieces. Absorbance (280 nm) and/or Rayleigh interference (655 nm) scans were collected at approximately 3 min intervals. Data analysis was performed with SEDFIT (v.15.01c) using a continuous c(s) distribution model as described previously^50^, scan file time-stamps were corrected^51^ and good fits were obtained with root mean squared deviation (r.m.s.d.) values corresponding to typical instrument noise values.

To analyse the aggregation of BTN3A1 B30.2 and BTN2A1 B30.2 in different concentrations, the sample contained BTN3A1 B30.2, BTN2A1 B30.2 (1 mg ml^−1^ or 5 mg ml^−1^ for each protein) and HMBPP (120 µM or 1.2 mM).

### AFM-SCFS analysis

The AFM-SCFS analysis procedure was performed using the JPK CellHesion unit as previously described^52^, and closely followed our previously reported protocol for APCs^4^. In brief, here MIA PaCa-2 cells (1 × 10^4^) were cultured on glass disks (containing 10% FBS, 1% penicillin–streptomycin and 95% humidity in DMEM) overnight at 37 °C and 5% CO_2_. After 4 h pretreatment with different compounds (1 µM HMBPP, 30 µM HMBPP-08 or 10 µM Zol), the glass disks were washed twice with PBS. The glass disks were quickly transferred to an AFM adapter with immediate addition of 1 ml of fresh medium (DMEM: 10% FBS and 1% penicillin–streptomycin). The AFM cantilever carrying the Vγ9Vδ2 T cell was lowered to the surface of an individual MIA PaCa-2 cell at a constant force of 500 pN and, after contact for 10 s, the cantilevers were withdrawn and the deformation coefficient was used to calculate the intercellular force. At least 10 groups of MIA PaCa-2/Vγ9Vδ2 T cell interaction curves were collected for each experiment. The raw data were normalized to the background readings. Blank readings for each set of affinity measurements (Vγ9Vδ2 T cells touching the glass surface) were collected immediately before each assay to perform background subtraction. All data were analysed using the JPK image processing software.

To measure the ability of HMBPP-15 in reducing the binding force between a Vγ9Vδ2 T cell and a MIA PaCa-2 cell, the MIA PaCa-2 cells were pretreated with HMBPP-15 (30 µM) for 4 h and the glass disks were washed twice to remove HMBPP-15. MIA PaCa-2 cells were then treated with zoledronate (10 µM) for 8 h. The following steps were the same as described above.

### TET labelling of BTN3A1 B30.2 and ^19^F-NMR experiments

The labelling of BTN3A1 B30.2 with TET (Sigma-Aldrich) was performed based on the reported protocols^53^. In brief, 100 µM BTN3A1 B30.2 and 1 mM 3,3,3-phosphinetriyltripropanoic acid hydrochloride (TCEP) (Bidepharmatech) were added to 100 µl buffer (20 mM HEPES pH 7.5, 150  mM NaCl) and incubated for 30 min at 4 °C. The solution was then passed through a pre-equilibrated PD-10 desalt column (GE Healthcare) to remove TCEP. After treatment with 1 mM 4-4′-dithiopydine (Aldrithiol) for 1 h at 4 °C, the buffer was exchanged to remove excess 4-DPS. The mixture was incubated with 1 mM TET for 4 h at 4 °C and was passed through a pre-equilibrated PD-10 desalt column to remove TET. All of the NMR samples were prepared in 20 µM in NMR buffer (20 mM HEPES pH 7.5, 150 mM NaCl, 10% D_2_O and 0.01% TFA). All NMR spectra were recorded on the 600 MHz Bruker (AV-HD-600X) system.

### Molecular dynamics simulations

The crystal structures of HMBPP- and DMAPP-bound complexes were prepared using the Protein Preparation Wizard (Schrödinger, release 2021-1) using the default settings. The apo complex was built by removing the HMBPP ligand after the preparation. All three systems were immersed in an orthorhombic SPC water box with 10 Å of buffer width and neutralized by randomly placing sodium ions in the water box. The prepared simulation systems were relaxed and simulated at 300 K and 1 atm using Desmond and OPLS4 force field (Schrödinger, release 2021-1). We simulated HMBPP and DMAPP bound to the BTN3A1–BTN2A1 complex as well as the apo form of the BTN3A1–BTN2A1 complex for 100 ns.

### FEP+

The relative binding-affinity values of the pyrophosphate series (compounds **1**–**6** in Extended Data Fig. 5a) were predicted using FEP+^54^ (Schrödinger, release 2023-1) using the default parameters. In brief, the compounds were prepared using Ligprep (Schrödinger, release 2023-1) and the compounds were docked into the prepared complex structure of the HMBPP-bound BTN2A1–BTN3A1 using core constraints based on a maximum common substructure. The crystal waters that clashed with docked ligands were removed. The simulations lasted 5 ns per lambda window, and each perturbation was distributed over 12 lambda windows.

### Computational modelling

The full-length BTN2A1 and BTN3A1 each comprise an ectodomain and an intracellular domain, connected through a coiled-coil segment. The coiled-coil domains of BTN2A1 and BTN3A1 were predicted using the CCFold web server (https://pharm.kuleuven.be/apps/biocryst/ccfold.php)^55^. The models were prepared using the Protein Preparation Wizard program (Schrödinger, release 2021-2). The protonation states of ionizable residues were predicted using PROPKA^56^ and the optimal hydrogen bonding networks were exhaustively sampled. Disulfide bridges were introduced between two Cys247 residues and between two Cys265 residues across the coiled-coil domains using the Cysteine Mutation workflow in BioLuminate (Schrödinger, release 2022-2).

### Extracellular BTN2A1–TCR interactions

The homology model of the BTN2A1 dimer ectodomain was built using the crystal structure of the BTN3A1 ectodomain (PDB: 4F80) as a template. The crystal structure of the Vγ9Vδ2 domain (PDB: 1HXM) was prepared using the Protein Preparation Wizard (Schrödinger, release 2021-2). The variable domain was docked to the BTN2A1 dimer ectodomain using PIPER^36^ (Schrödinger, release 2021-3). We applied attractive restraints on Arg65, Arg124, Tyr126 and Glu135 on the IgV domain and Arg20, Glu70 and His85 on the Vγ9 domain based on mutation data reported earlier^22,23^. Seven poses were selected and each pose was allowed to relax in a molecular dynamics simulation of 50 ns using the default settings in Desmond (Schrödinger, release 2021-3). In brief, each pose was first solvated in an orthorhombic SPC water box with 10 Å buffer distance in all directions in Desmond. The default relaxation protocol, consisting of a series of minimizations and short simulations at low temperature with various restraints on the solute, was applied to each system before the start of the production NPT simulations at 300 K and 1 atm. The timestep used in the RESPA integrator^57^ was 2 fs for bonded and near and 6 fs for far. A Nose–Hoover chain thermostat^58^ with a 1 ps relaxation time and a Martyna–Tobias–Klein barostat^59^ with 2 ps relaxation time and isotropic coupling were used to maintain the temperature and pressure. One of the poses was selected based on its low r.m.s.d. drift during the simulation, its lack of charge repulsion in the trajectory and its reasonable interactions.

### Extracellular BTN2A1–BTN3A1 interactions

We initially assumed that the BTN3A1-binding site on BTN2A1 does not overlap BTN2A1’s interaction epitope for TCR, so we docked the BTN3A1 ectodomain from PDB 4F80 to the modelled TCR–BTN2A1 ectodomain complex using the PIPER program and attractive restraints were applied to Tyr127, Tyr134 and Gln129 on BTN3A1 on the basis of mutation data^23^. Poses, wherein the BTN3A1 and BTN2A1 ectodomains were in near antiparallel orientations, were filtered out, as were poses in which the restraints were not satisfied. The filtering process produced only one pose. The interacting monomers of BTN3A1 and BTN2A1 were extracted from the docking pose and solvated in a water box with seven sodium ions to neutralize the system. The solvated system was simulated using the default settings in Desmond as described above. As there was only one pose, we allowed the simulation to continue for 1 μs. The frames from the last 200 ns were clustered on the basis of Cα r.m.s.d. and the cluster representative from the largest cluster was used for building the full-length model.

### A visualization of the whole inside-out signalling based on computational modelling

The BTN2A1 B30.2 dimer was extracted from PDB 8JYE and the BTN3A1 B30.2 dimer was extracted from PDB 5ZXK. The BTN3A1 ectodomain dimer was taken from PDB 4F80. The individual full-length models of BTN3A1 and BTN2A1 were manually built by connecting the ectodomain complex, the coiled-coil segment and the intracellular domains, followed by minimization of the linkage residues between the connected domains (with this minimization carried out in implicit solvation^60^ using the default settings in Prime (Schrödinger, release 2022-2)). The minimization started with a conjugated gradient method followed by a truncated Newton method, and was performed in two iterations with 65 steps per iteration. The inactive complex model was generated by aligning full-length models to the last frame of the 1 µs simulation of the BTN2A1–BTN3A1 docking pose. The active complex model was generated by aligning the B30.2 domains of the full-length models to a crystal structure of the BTN2A1–BTN3A1 B30.2 complex (PDB: 8JYE). The TCR was added to the model by aligning the BTN2A1 ectodomain in the docked pose of TCR–BTN2A1 complex to the active complex model, and manual tilting was introduced to the BTN3A1 ectodomain to resolve any clashes. Finally, complete models were minimized using the default settings.

### Quantitative model for Vγ9Vδ2 T cell activation by phosphoantigens

For the 13 library compounds, a quantitative model for their activities for sensitizing MIA PaCa-2 cells to Vγ9Vδ2 T cell killing (EC_50_) using a partial least-square method to regress binding affinities to the preconditioned BTN2A1–BTN3A1 complexes (*K*_D_) and ClogP, against the cell pEC_50_ results. That is: pEC_50_(predicted) = *a*·p*K*_D_ + *b*·ClogP + *c*, where pEC_50_ is −log_10_[EC_50_], p*K*_D_ is −log_10_[*K*_D_], and *a*–*c* are coefficients. EC_50_ values were determined using the cell killing assays; *K*_D_ values were determined by ITC and ClogP values were calculated using Chemdraw.

### Synthetic aspects

HMBPP (compound **1**), DMAPP (compound **2**), IPP (compound **3**) and HMBPP analogues were synthesized as described previously^61–63^.

#### (*E*)-4-hydroxy-3-methylbut-2-en-1-yl diphosphate (**1**, HMBPP)

^1^H NMR (400 MHz, D_2_O) δ ppm 5.63 (t, *J* = 6.8 Hz, 1H), 4.50 (dd, *J*_1_ = *J*_2_ = 7.2 Hz, 2H), 3.99 (s, 2H), 1.68 (s, 3H); ^31^P NMR (162 MHz, D_2_O) δ ppm −6.56 (d, *J* = 20.0 Hz, 1P), −10.38 (d, *J* = 20.0 Hz, 1P). HRMS (*m*/*z*) [M-H]^+^ calculated 260.9923, found 260.9929.

#### 3-methylbut-2-en-1-yl diphosphate (**2**, DMAPP)

^1^H NMR (400 MHz, D_2_O) δ ppm 5.43 (t, *J* = 7.24 Hz, 1H), 4.44 (t, *J* = 6.88 Hz, 2H), 1.75 (s, 3H), 1.70 (s, 3H); ^31^P NMR (162 MHz, D_2_O) δ ppm −8.41 (d, *J* = 21.38 Hz, 1P), −10.55 (d, *J* = 21.38 Hz, 1P). HRMS (*m*/*z*) [M-H]^+^ calculated 244.9980, found 244.9978.

#### 3-methylbut-3-en-1-yl diphosphate (**3**, IPP)

^1^H NMR (400 MHz, D_2_O) δ ppm 4.82 (m, 2H), 4.04 (t, *J* = 6.70 Hz, 2H), 2.38 (t, *J* = 6.7 Hz, 2H), 1.75 (s, 3H); ^31^P NMR (162 MHz, D_2_O) δ ppm −9.49 (d, *J* = 20.90 Hz, 1P), −10.79 (d, *J* = 20.9 Hz, 1P). HRMS (*m*/*z*) [M-H]^+^ calculated 244.9980, found 244.9981.

#### (*E*)-4-hydroxy-3-ethyl-but-2-enyl diphosphate (**4**)

^1^H NMR (400 MHz, D_2_O) δ ppm 5.55 (t, *J* = 6.80 Hz, 1H), 4.56 (dd, *J*_1_ = *J*_2_ = 7.20 Hz, 2H), 4.01 (s, 2H), 2.07 (q, *J* = 7.60 Hz, 2H), 0.92 (t, *J* = 7.60 Hz,3H); ^31^P NMR (162 MHz, D_2_O) δ ppm −8.66 (d, *J* = 19.44 Hz, 1P), −10.57 (d, *J* = 19.44 Hz, 1P). HRMS (*m*/*z*) [M-H]^+^ calculated 275.0086, found 275.0086.

#### (*E*)-3-(hydroxymethyl)hexa-2,5-dienyl diphosphate (**5**)

^1^H NMR (400 MHz, D_2_O) δ ppm 5.91–5.87 (m, 1H), 5.64 (t, *J* = 6.80 Hz, 1H), 5.16–5.10 (m, 2H), 4.54 (dd, *J*_1_ = *J*_2_ = 6.80 Hz, 2H), 4.12 (s, 2H), 2.91 (d, *J* = 6.40 Hz, 2H); ^31^P NMR (162 MHz, D_2_O) δ ppm −9.17 (d, *J* = 21.1 Hz, 1P), −10.66 (d, *J* = 21.1 Hz, 1P). HRMS (*m*/*z*) [M-H]^+^ calculated 287.0081, found 287.0086.

#### (*E*)-4-hydroxy-3-methylpent-2-en-1-yl diphosphate (**6**)

^1^H NMR (400 MHz, D_2_O) δ ppm 5.61 (t, *J* = 6.64 Hz, 1H), 4.48 (t, *J* = 6.96 Hz, 2H), 4.23 (q, *J* = 6.60 Hz, 1H), 1.65 (s, 3H), 1.21 (d, *J* = 6.52 Hz, 3H); ^31^P NMR (162 MHz, D_2_O) δ ppm −8.07 (d, *J* = 21.06 Hz, 1P), −10.51 (d, *J* = 21.06 Hz, 1P). HRMS (*m*/*z*) [M-H]^+^ calculated 275.0086, found 275.0095.

#### (*E*)-((hydroxy((4-hydroxy-3-methylbut-2-en-1-yl)oxy)phosphoryl)methyl)phosphonic acid (**7**)

^1^H NMR (400 MHz, D_2_O) δ ppm 5.63 (t, *J* = 6.64 Hz, 1H), 4.49 (t, *J* = 7.36 Hz, 2H), 4.02 (s, 2H), 2.15 (t, *J* = 19.76 Hz, 2H), 1.71 (s, 3H). ^31^P NMR (162 MHz, D_2_O) δ ppm 18.37 (d, *J* = 9.56 Hz, 1P), 15.06 (d, *J* = 9.56 Hz, 1P). HRMS (*m*/*z*) [M-H]^+^ calculated 259.0137, found 259.0137.

#### (*E*)-(dichloro(hydroxy((4-hydroxy-3-methylbut-2-en-1yl)oxy)phosphoryl)methyl diphosphate (**8**)

^1^H NMR (400 MHz, D_2_O) δ ppm 5.55 (t, *J* = 6.2 Hz, 1H), 4.60 (dd, *J*_1_= *J*_2_ = 7.4 Hz, 2H), 3.90 (s, 2H), 1.59 (s, 3H). ^31^P NMR (162 MHz, D_2_O) δ ppm 10.92 (d, *J* = 9.0 Hz, 1P), 8.07 (d, *J* = 9.0 Hz, 1P). HRMS (*m*/*z*) [M-H]^+^ calculated 326.9357, found 326.9359.

#### (*E*)-(chloro(hydroxy((4-hydroxy-3-methylbut-2-en-1-yl)oxy)phosphoryl)methyl)phosphonic acid (**9**)

^1^H NMR (400 MHz, D_2_O) δ ppm 5.63 (t, *J* = 6.2 Hz, 1H), 4.56 (dd, *J*_1_ = *J*_2_ = 7.4 Hz, 2H), 4.0 (s, 2H), 3.8 (dd, *J*_1_ = *J*_2_ = 15.4 Hz, 1H), 1.70 (s, 3H). ^31^P NMR (162 MHz, D_2_O) δ ppm 13.99 (d, *J* = 9.0 Hz, 1P), 9.31 (d, *J* = 9.0 Hz, 1P). HRMS (*m*/*z*) [M-H]^+^ calculated 345.0667, found 345.0669.

#### (*E*)-(difluoro(hydroxy((4-hydroxy-3-methylbut-2-en-1-yl)oxy)phosphory)methyl)phosphonic acid (**10**)

^1^H NMR (400 MHz, D_2_O) δ ppm 5.61 (t, *J* = 6.4 Hz, 1H), 4.58 (dd, *J*_1_= *J*_2_ = 7.28 Hz, 2H), 3.99 (s, 2H), 1.68 (s, 3H). ^31^P NMR (162 MHz, D_2_O) δ ppm 12.86 (m, 1P), 7.52 (m, 1P). HRMS (*m*/*z*) [M-H]^+^ calculated 294.9948, found 294.9952.

#### (*E*)-(fluoro(hydroxy((4-hydroxy-3-methylbut-2-en-1-yl)oxy)phosphoryl)methyl)phosphonic acid (**11**)

^1^H NMR (400 MHz, D_2_O) δ ppm 5.61 (t, *J* = 6.6 Hz, 1H), 4.64 (dd, *J*_1_= *J*_2_ = 12.2 Hz, 1H), 4.54 (dd, *J*_1_= *J*_2_ = 7.28 Hz, 2H), 3.98 (s, 2H), 1.68 (s, 3H). ^31^P NMR (162 MHz, D_2_O) δ ppm 12.86 (dd, *J*_1_ = 11.2 Hz, *J*_2_ = 62.5 Hz, 1P), 7.52 (dd, *J*_1_ = 11.2 Hz, *J*_2_ = 58.0 Hz, 1P). HRMS (*m*/*z*) [M-H]^+^ calculated 277.0167, found 277.0169.

#### (*E*)-3-(hydroxymethyl)undec-2-enyl diphosphate (HMBPP-05)

^1^H NMR (400 MHz, D_2_O) δ ppm 5.64 (t, *J* = 6.12 Hz, 1H), 4.55 (dd, *J*_1_ = *J*_2_ = 6.72 Hz, 2H), 4.06 (s, 2H), 2.13 (t, *J* = 7.32 Hz, 2H), 1.39–1.28 (m, 14H), 0.85 (t, *J* = 6.12 Hz,3H); ^31^P NMR (162 MHz, D_2_O) δ ppm −10.5 (d, *J* = 19.44 Hz, 1P), −10.8 (d, *J* = 19.44 Hz, 1P). HRMS (*m*/*z*) [M-H]^+^ calculated 359.1021, found 359.1025.

#### (*E*)-4-hydroxy-3-(4-methylbenzyl)but-2-enyl diphosphate (HMBPP-08)

^1^H NMR (400 MHz, D_2_O) δ ppm 7.09–7.07 (m, 4H), 5.75 (t, *J* = 6.80 Hz, 1H), 4.59 (dd, *J*_1_ = *J*_2_ = 6.40 Hz, 2H), 3.85 (s, 2H), 3.37 (s, 2H), 2.18 (s, 3H); ^31^P NMR (162 MHz, D_2_O) δ ppm −7.61 (d, *J* = 21.10 Hz, 1P), −10.44 (d, *J* = 21.10 Hz, 1P). HRMS (*m*/*z*) [M-H]^+^ calculated 351.0397, found 351.0399.

#### (*E*)-4-([1,1′-biphenyl]-4-yl)-3-(hydroxymethyl)but-2-en-1-yl diphosphate (HMBPP-15)

^1^H NMR (400 MHz, D_2_O) δ 7.66 (dd, *J* = 17.6, 7.8 Hz, 4H), 7.51 (t, *J* = 7.6 Hz, 2H), 7.40 (dd, *J* = 16.6, 7.8 Hz, 3H), 5.88 (t, *J* = 6.7 Hz, 1H), 4.75–4.66 (m, 2H), 4.00 (s, 2H), 3.59 (s, 2H). ^31^P NMR (162 MHz, D_2_O) δ −10.56 (d, *J* = 21.0 Hz, 1P), −8.55 (d, J = 21.0 Hz, 2P); HRMS (*m*/*z*) [M-H]^+^ calculated 413.0555, found 413.0545.

### Statistical analysis

For comparison of more than two independent groups, we used either Brown–Forsythe and Welch ANOVA with Dunnett’s T3 multiple-comparison test or Kruskal–Wallis tests with a Dunn’s multiple-comparison test. For comparison of two paired groups, two-way ANOVA with Dunnett’s multiple-comparison test was used. All data were analysed using GraphPad Prism v.9.0 (GraphPad) and are expressed as mean ± s.e.m.

### Reporting summary

Further information on research design is available in the Nature Portfolio Reporting Summary linked to this article.

## Online content

Any methods, additional references, Nature Portfolio reporting summaries, source data, extended data, supplementary information, acknowledgements, peer review information; details of author contributions and competing interests; and statements of data and code availability are available at 10.1038/s41586-023-06525-3.

### Supplementary information

Supplementary Information Supplementary Figs. 1–11 and Supplementary Tables 1–5.

Reporting Summary

### Source data

Source Data Fig. 1

Source Data Fig. 2

Source Data Fig. 3

Source Data Fig. 5

Source Data Fig. 6

Source Data Extended Data Fig. 1

Source Data Extended Data Fig. 2

Source Data Extended Data Fig. 3

Source Data Extended Data Fig. 6

Source Data Extended Data Fig. 7

## Figures and Tables

**Extended Data Table 1:**
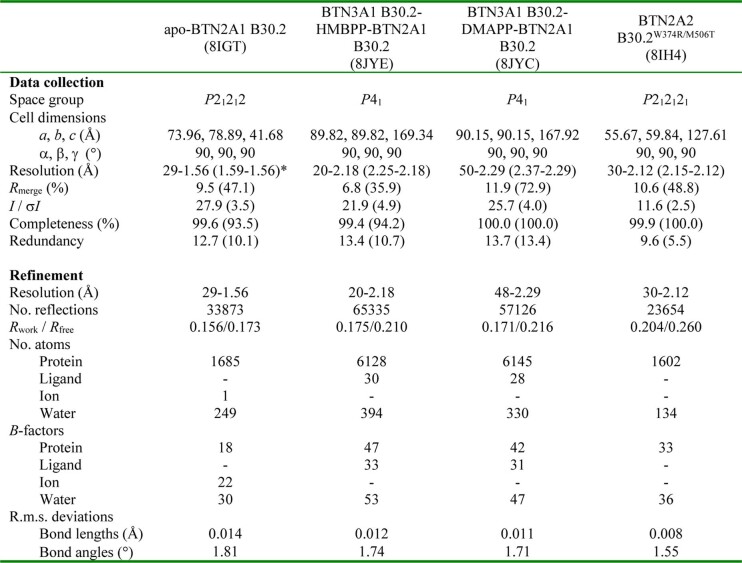
X-ray data collection and refinement statistics of human butyrophilins intracellular B30.2 domain and the complexes

For each structure one crystal was used.

*Values in parentheses are for highest-resolution shell.

**Extended Data Table 2:**
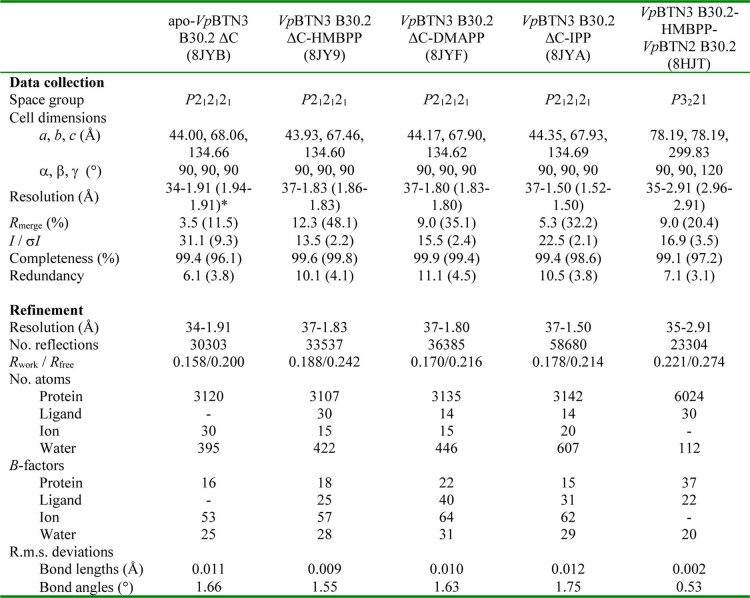
X-ray data collection and refinement statistics of alpaca butyrophilins intracellular domain and the complexes

For each structure one crystal was used.

*Values in parentheses are for highest-resolution shell.

**Extended Data Table 3:**
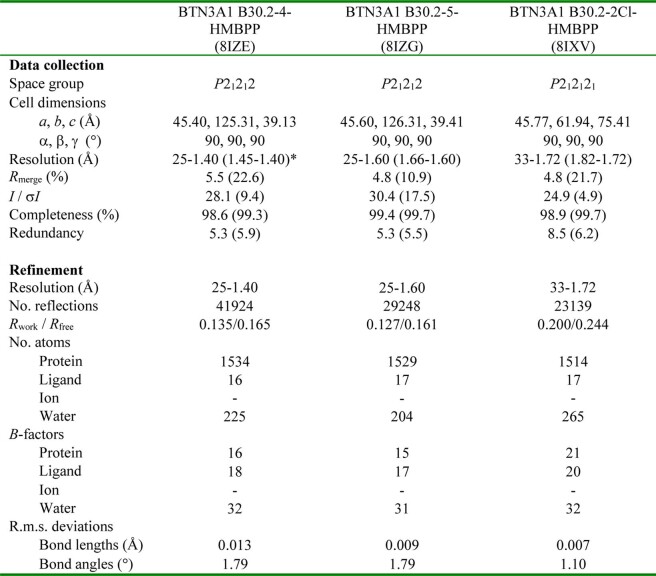
X-ray data collection and refinement statistics of human BTN3A1 B30.2 domain with compound 4, 5, and 8

For each structure one crystal was used.

*Values in parentheses are for highest-resolution shell.

## Data Availability

Any additional information required to reanalyse the data are publicly available. The crystal structures are deposited at the PDB: 8IGT, 8JYE, 8JYC, 8IH4, 8JYB, 8JY9, 8JYF, 8JYA, 8HJT, 8IZE, 8IZG and 8IXV. The structure data from the following PDB accessions were used: 5ZXK, 4F80, 1HXM, 4V1P and 6J06. Source data are provided with this paper.

## References

[1] Sandstrom A (2014). The intracellular B30.2 domain of butyrophilin 3A1 binds phosphoantigens to mediate activation of human Vγ9Vδ2 T cells. Immunity.

[2] Rhodes DA (2015). Activation of human γδ T cells by cytosolic interactions of BTN3A1 with soluble phosphoantigens and the cytoskeletal adaptor periplakin. J. Immunol..

[3] Sebestyen Z (2016). RhoB mediates phosphoantigen recognition by Vγ9Vδ2 T cell receptor. Cell Rep..

[4] Yang Y (2019). A structural change in butyrophilin upon phosphoantigen binding underlies phosphoantigen-mediated Vγ9Vδ2 T cell activation. Immunity.

[5] Rossjohn J (2015). T cell antigen receptor recognition of antigen-presenting molecules. Annu. Rev. Immunol..

[6] Vantourout P, Hayday A (2013). Six-of-the-best: unique contributions of γδ T cells to immunology. Nat. Rev. Immunol..

[7] Goldstein JL, Brown MS (1990). Regulation of the mevalonate pathway. Nature.

[8] Tanaka Y (1995). Natural and synthetic non-peptide antigens recognized by human γδ T cells. Nature.

[9] Gober HJ (2003). Human T cell receptor γδ cells recognize endogenous mevalonate metabolites in tumor cells. J. Exp. Med..

[10] Hintz M (2001). Identification of (*E*)-4-hydroxy-3-methyl-but-2-enyl pyrophosphate as a major activator for human γδ T cells in *Escherichia coli*. FEBS Lett..

[11] Zhao L, Chang WC, Xiao Y, Liu HW, Liu P (2013). Methylerythritol phosphate pathway of isoprenoid biosynthesis. Annu. Rev. Biochem..

[12] Palakodeti A (2012). The molecular basis for modulation of human Vγ9Vδ2 T cell responses by CD277/butyrophilin-3 (BTN3A)-specific antibodies. J. Biol. Chem..

[13] Harly C (2012). Key implication of CD277/butyrophilin-3 (BTN3A) in cellular stress sensing by a major human γδ T-cell subset. Blood.

[14] Wang H (2013). Butyrophilin 3A1 plays an essential role in prenyl pyrophosphate stimulation of human Vγ2Vδ2 T cells. J. Immunol..

[15] Hsiao CH (2014). Synthesis of a phosphoantigen prodrug that potently activates Vγ9Vδ2 T-lymphocytes. Chem. Biol..

[16] Kabelitz, D., Lettau, M. & Janssen, O. Immunosurveillance by human γδ T lymphocytes: the emerging role of butyrophilins. *F1000Res*. **6**(F1000 Faculty Rev), 782 (2017). 10.12688/f1000research.11057.1 PMC5464295 28649364

[17] Vavassori S (2013). Butyrophilin 3A1 binds phosphorylated antigens and stimulates human γδ T cells. Nat. Immunol..

[18] Gu S (2017). Phosphoantigen-induced conformational change of butyrophilin 3A1 (BTN3A1) and its implication on Vγ9Vδ2 T cell activation. Proc. Natl Acad. Sci. USA.

[19] Wang H, Morita CT (2015). Sensor function for butyrophilin 3A1 in prenyl pyrophosphate stimulation of human Vγ2Vδ2 T cells. J. Immunol..

[20] Castella B (2017). The ATP-binding cassette transporter A1 regulates phosphoantigen release and Vγ9Vδ2 T cell activation by dendritic cells. Nat. Commun..

[21] Vantourout P (2018). Heteromeric interactions regulate butyrophilin (BTN) and BTN-like molecules governing γδ T cell biology. Proc. Natl Acad. Sci. USA.

[22] Rigau M (2020). Butyrophilin 2A1 is essential for phosphoantigen reactivity by γδ T cells. Science.

[23] Karunakaran MM (2020). Butyrophilin-2A1 directly binds germline-encoded regions of the Vγ9Vδ2 TCR and is essential for phosphoantigen sensing. Immunity.

[24] Melandri D (2018). The γδTCR combines innate immunity with adaptive immunity by utilizing spatially distinct regions for agonist selection and antigen responsiveness. Nat. Immunol..

[25] Willcox CR (2019). Butyrophilin-like 3 directly binds a human Vγ4^+^ T cell receptor using a modality distinct from clonally-restricted antigen. Immunity.

[26] Yuan, L. et al. Phosphoantigens are molecular glues that promote butyrophilin 3A1/2A1 association leading to Vγ9Vδ2 T cell activation. Preprint at *BioRxiv*10.1101/2022.01.02.474068 (2022).

[27] Hsiao CC, Nguyen K, Jin Y, Vinogradova O, Wiemer AJ (2022). Ligand-induced interactions between butyrophilin 2A1 and 3A1 internal domains in the HMBPP receptor complex. Cell Chem. Biol..

[28] D’Cruz AA, Babon JJ, Norton RS, Nicola NA, Nicholson SE (2013). Structure and function of the SPRY/B30.2 domain proteins involved in innate immunity. Protein Sci..

[29] Perfetto L (2013). Exploring the diversity of SPRY/B30.2-mediated interactions. Trends Biochem. Sci..

[30] Roelofs AJ (2009). Peripheral blood monocytes are responsible for γδ T cell activation induced by zoledronic acid through accumulation of IPP/DMAPP. Br. J. Haematol..

[31] Fichtner AS (2020). Alpaca (*Vicugna pacos*), the first nonprimate species with a phosphoantigen-reactive Vγ9Vδ2 T cell subset. Proc. Natl Acad. Sci. USA.

[32] Belmant C (2000). A chemical basis for recognition of nonpeptide antigens by human γδ T cells. FASEB J..

[33] Salim M (2017). BTN3A1 discriminates γδ T cell phosphoantigens from nonantigenic small molecules via a conformational sensor in its B30.2 domain. ACS Chem. Biol..

[34] Nguyen K (2017). The butyrophilin 3A1 intracellular domain undergoes a conformational change involving the juxtamembrane region. FASEB J..

[35] Willcox CR (2023). Phosphoantigen sensing combines TCR-dependent recognition of the BTN3A IgV domain and germline interaction with BTN2A1. Cell Rep..

[36] Kozakov D, Brenke R, Comeau SR, Vajda S (2006). PIPER: an FFT-based protein docking program with pairwise potentials. Proteins.

[37] Karunakaran, M. M. et al. Division of labor and cooperation between different butyrophilin proteins controls phosphoantigen-mediated activation of human γδ T cells. Preprint at *Research Square*10.21203/rs.3.rs-2583246/v1 (2023).

[38] Dieli, F., Fadda, R. & Caccamo, N. Butyrophilin 3A1 presents phosphoantigens to human γδ T cells: the fourth model of antigen presentation in the immune system. *Cell Mol. Immunol.* **11**, 123–125 (2014). 10.1038/cmi.2013.54 PMC4003376 24270471

[39] Gassart AD (2021). Development of ICT01, a first-in-class, anti-BTN3A antibody for activating Vγ9Vδ2 T cell-mediated antitumor immune response. Sci. Trans. Med..

[40] Arnett HA, Viney JL (2014). Immune modulation by butyrophilins. Nat. Rev. Immunol..

[41] Di Marco Barros R (2016). Epithelia use butyrophilin-like molecules to shape organ-specific γδ T cell compartments. Cell.

[42] Collaborative Computational Project, Number 4 (1994). The CCP4 suite: programs for protein crystallography. Acta. Crystallogr. D.

[43] Emsley P, Cowtan K (2004). Coot: model-building tools for molecular graphics. Acta. Crystallogr. D.

[44] Murshudov GN (2011). REFMAC5 for the refinement of macromolecular crystal structures. Acta. Crystallogr. D.

[45] Sanjana NE, Shalem O, Zhang F (2014). Improved vectors and genome-wide libraries for CRISPR screening. Nat. Methods.

[46] Shalem O (2014). Genome-scale CRISPR-Cas9 knockout screening in human cells. Science.

[47] Patel SJ (2017). Identification of essential genes for cancer immunotherapy. Nature.

[48] Li W (2014). MAGeCK enables robust identification of essential genes from genome-scale CRISPR/Cas9 knockout screens. Genome Biol..

[49] Zhao, H., Brautigam, C. A., Ghirlando, R. & Schuck, P. Overview of current methods in sedimentation velocity and sedimentation equilibrium analytical ultracentrifugation. *Curr. Protoc. Protein Sci.* Unit 20.12 (2013). 10.1002/0471140864.ps2012s71 PMC3652391 23377850

[50] Schuck P, Perugini MA, Gonzales NR, Howlett GJ, Schubert D (2002). Size-distribution analysis of proteins by analytical ultracentrifugation: strategies and application to model systems. Biophysical.

[51] Zhao H (2013). Recorded scan times can limit the accuracy of sedimentation coefficients in analytical ultracentrifugation. Anal. Biochem..

[52] Flach TL (2011). Alum interaction with dendritic cell membrane lipids is essential for its adjuvanticity. Nat. Med..

[53] Liu JJ, Horst R, Katritch V, Stevens RC, Wüthrich K (2012). Biased signaling pathways in β2-adrenergic receptor characterized by ^19^F-NMR. Cell.

[54] Wang L (2015). Accurate and reliable prediction of relative ligand binding potency in prospective drug discovery by way of a modern free-energy calculation protocol and force field. J. Am. Chem. Soc..

[55] Guzenko D, Strelkov SV (2018). CCFold: rapid and accurate prediction of coiled-coil structures and application to modelling intermediate filaments. Bioinformatics.

[56] Olsson MHM, Søndergaard CR, Rostkowski M, Jensen JH (2011). PROPKA3: consistent treatment of internal and surface residues in empirical p*K*_a_ predictions. J. Chem. Theory Comput..

[57] Tuckerman M, Berne BJ, Martyna GJ (1992). Reversible multiple time scale molecular dynamics. J. Chem. Phys..

[58] Martyna GJ, Klein ML (1992). Nose-Hoover chain: the canonical ensemble via continuous dynamics. J. Chem. Phys..

[59] Martyna GJ, Tobias DJ, Klein ML (1994). Constant pressure molecular dynamics algorithm. J. Chem. Phys..

[60] Li J (2011). The VSGB 2.0 model: a next generation energy model for high resolution protein structure modeling. Proteins.

[61] Davisson VJ (1986). Phosphorylation of isoprenoid alcohols. J. Org. Chem..

[62] Hecht S, Amslinger S, Jauch J, Kis K, Rohdich F (2002). Studies on the non-mevalonate isoprenoid biosynthetic pathway. Simple methods for preparation of isotope-labeled (*E*)-1-hydroxy-2-methylbut-2-enyl 4-diphosphate. Tetrahedron Lett..

[63] Reichenberg A (2003). Replacing the pyrophosphate group of HMB-PP by a diphosphonate function abrogates its potential to activate human γδ T cells but does not lead to competitive antagonism. Bioorg. Med. Chem. Lett..

