# Supplementary material for: Phosphoantigens glue butyrophilin 3A1 and 2A1 to activate Vγ9Vδ2 T cells
Source: Nature. 2023 Sep 6;621(7980):840–8. doi: 10.1038/s41586-023-06525-3 (PMC10533412; doi:10.1038/s41586-023-06525-3)
Supplement: Supplementary file 1 — Supplementary Figs. 1–11 and Supplementary Tables 1–5. [file 41586_2023_6525_MOESM1_ESM.pdf]

nature portfolio

https://doi.org/10.1038/s41586-023-06525-3

Supplementary information

Phosphoantigens glue butyrophilin 3A1 and 2A1 to activate Vγ9Vδ2 T cells

In the format provided by the authors and unedited

Supplementary Information for

Phosphoantigens glue butyrophilin 3A1 and 2A1 to activate Vγ9Vδ2 T cells

Linjie Yuan[1]*, Xianqiang Ma[1]*, Yunyun Yang[1,2]*, Yingying Qu[1], Xin Li[1], Xiaoyu Zhu[3], Weiwei Ma[1], Jianxin Duan[4], Jing Xue[1], Haoyu Yang[1], Jian-Wen Huang[2], Simin Yi[2], Mengting Zhang[2], Ningning Cai[1], Lin Zhang[1], Qingyang Ding[1], Kecheng Lai[2], Chang Liu[2], Lilan Zhang[2], Xinyi Liu[5], Yirong Yao[6], Shuqi Zhou[6], Xian Li[2], Panpan Shen[2], Qing Chang[6,7], Satish R. Malwal[8], Yuan He[9], Wenqi Li[6,7], Chunlai Chen[6], Chun-Chi Chen[2], Eric Oldfield[8], Rey-Ting Guo[2†], Yonghui Zhang[1†]

[1] Tsinghua-Peking Center for Life Sciences, State Key Laboratory of Membrane Biology, School of Pharmaceutical Sciences, Tsinghua University; Beijing 100084, China.

[2] State Key Laboratory of Biocatalysis and Enzyme Engineering, Hubei Hongshan Laboratory, Hubei Collaborative Innovation Center for Green Transformation of Bio-Resources, Hubei Key Laboratory of Industrial Biotechnology, School of Life Sciences, Hubei University, Wuhan 430062, China.

[3] Department of Hematology, The First Affiliated Hospital of USTC, Division of Life Sciences and Medicine, University of Science and Technology of China, Hefei, 230001, P.R. China.

[4] Schrödinger, GmbH, Glücksteinallee 25, Mannheim, Germany.

[5] School of Medicine, Tsinghua University, Beijing, 100084, China.

[6] School of Life Sciences, Tsinghua University, Beijing, 100084, China.

[7] Beijing Advanced Innovation Center for Structural Biology, Technology Center for Protein Sciences, Tsinghua University, Beijing 100084, China.

[8] Department of Chemistry, University of Illinois at Urbana-Champaign, Urbana, IL 61801, USA.

[9] Research Beyond Borders, Boehringer Ingelheim (China), Shanghai 200040, China.

*These authors contributed equally to this work.

[†]Corresponding author. Email: zhangyonghui@tsinghua.edu.cn (Y.Z.) (lead contact); guoreyting@hubu.edu.cn (R.-T.G.)

Tables of contents

Supplementary Figure 1	Electron density maps.
Supplementary Figure 2	The torsion angle frequency of the 2-methyl-butenyl group in IPP.
Supplementary Figure 3	Sequence alignment of human and alpaca BTN proteins.
Supplementary Figure 4	MD simulations and ^{19}F-NMR studies of 3A1 B30.2 fluctuations induced by pAg binding and upon association with 2A1 B30.2.
Supplementary Figure 5	Sequence alignment of the full-length 2A1 and 2A2 proteins.
Supplementary Figure 6	Gating strategy for flow cytometry analysis shown in Extended Data Fig. 2h.
Supplementary Figure 7	Gating strategy for flow cytometry analysis shown in Extended Data Fig. 3e.
Supplementary Figure 8	Gating strategy for flow cytometry analysis shown in Extended Data Fig. 6f.
Supplementary Figure 9	Gating strategy for flow cytometry analysis shown in Extended Data Fig. 7c.
Supplementary Figure 10	Gating strategy for flow cytometry analysis shown in Extended Data Fig. 7d.
Supplementary Figure 11	Gating strategy for flow cytometry analysis shown in Extended Data Fig. 7g.
Supplementary Table 1	The 2A1-3A1 interaction in the presence of HMBPP or DMAPP.
Supplementary Table 2	Interactions between 3A1 B30.2 and 2A1 B30.2.
Supplementary Table 3	Interactions between 2A1 A chain and B chain.
Supplementary Table 4	Summary of cell activities and binding affinities of HMBPP analogs.
Supplementary Table 5	List of primers used.

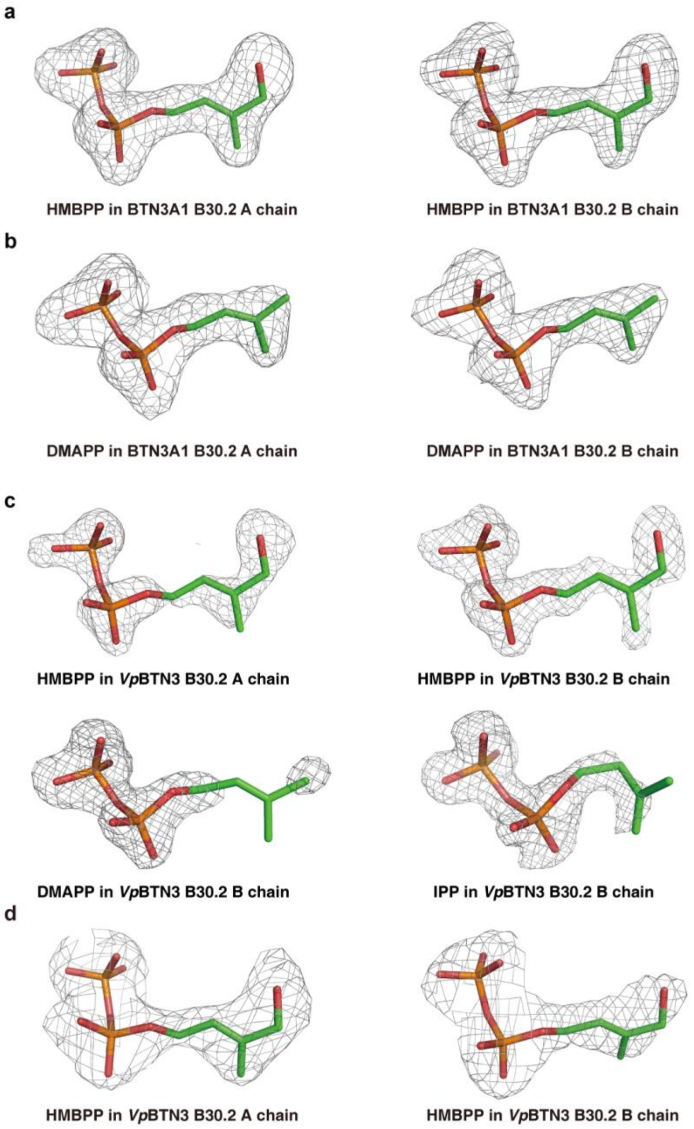

Supplementary Fig. 1| Electron density maps. a, Electron density maps of HMBPP in the 3A1 B30.2-HMBPP-2A1 B30.2 complex structure (PDB : 8JYE). The Fo-Fc omit maps of HMBPP in the A and B chains were each contoured at 2.5 σ (gray). **b,** Electron density maps of DMAPP in the 3A1 B30.2-DMAPP-2A1 B30.2 complex structure (PDB: 8JYC). The Fo-Fc omit maps of DMAPP in the A and B chains were each contoured at 2.5 σ (gray). **c,** Electron density maps of HMBPP in the *Vp*BTN3 B30.2-HMBPP complex structure (PDB: 8JY9); DMAPP in the *Vp*BTN3 B30.2-DMAPP complex structure (PDB: 8JYF); and IPP in the *Vp*BTN3 B30.2-IPP complex structure (PDB: 8JYA). The Fo-Fc omit maps of HMBPP in the A and B chains, DMAPP in the

B chain and IPP in the B chain were each contoured at 2.5 σ (gray). There is partial density loss of the alkyl side chain for both DMAPP and IPP, possibly owing to the lack of the stabilizing 1-OH group in HMBPP. **d,** Electron density maps of HMBPP in the *Vp*BTN3 B30.2-HMBPP-*Vp*BTN2 B30.2 complex structure (PDB: 8HJT). The Fo-Fc omit maps of HMBPP in the A and B chains were each contoured at 2.5 σ (gray).

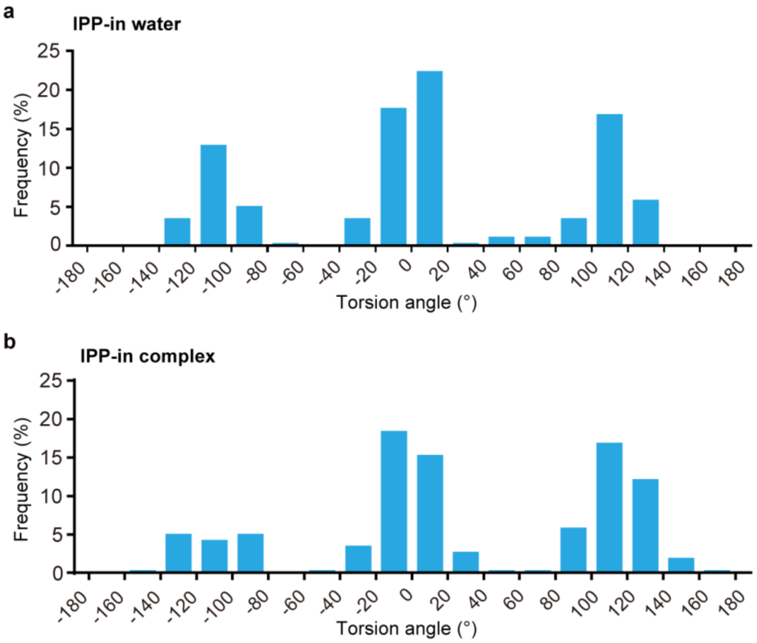

Supplementary Fig. 2| The torsion angle frequency of the 2-methyl-butenyl group in IPP. a, IPP alone in water solvent. **b,** When IPP is in the complex of 3A1 and 2A1 B30.2 domains.

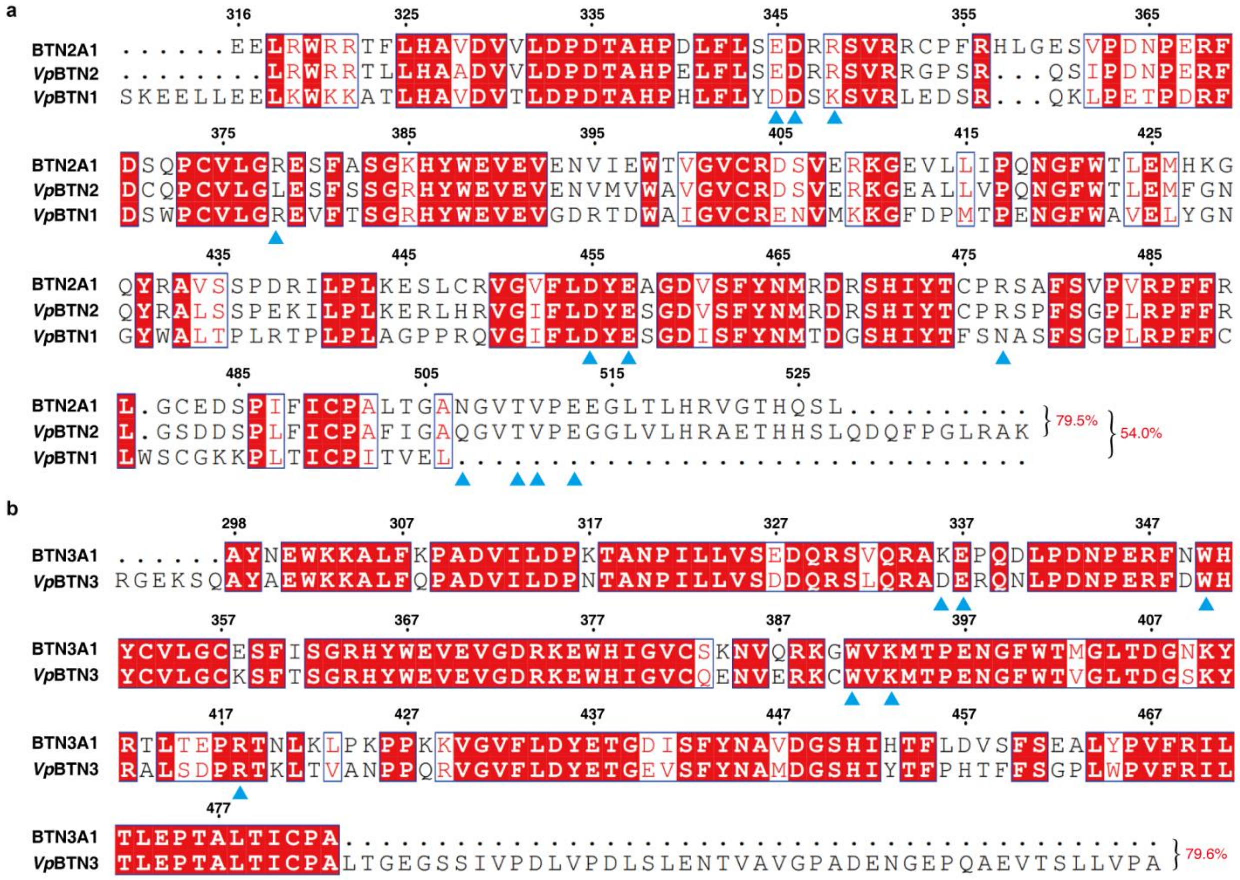

Supplementary Fig. 3| Sequence alignment of human and alpaca BTN proteins. a, Sequence alignment indicating 79.5% similarity between *Vp*BTN2 B30.2 and 2A1 B30.2, and 54.0% similarity between *Vp*BTN1 B30.2 and BTN2A1 B30.2. Blue triangles highlight the residues in 2A1 B30.2 that involved in the interaction with 3A1 B30.2. **b,** Sequence alignment indicating 79.6% similarity between *Vp*BTN3 and 3A1 B30.2. Blue triangles highlight the residues in 3A1 B30.2 that involved in the interaction with 2A1 B30.2.

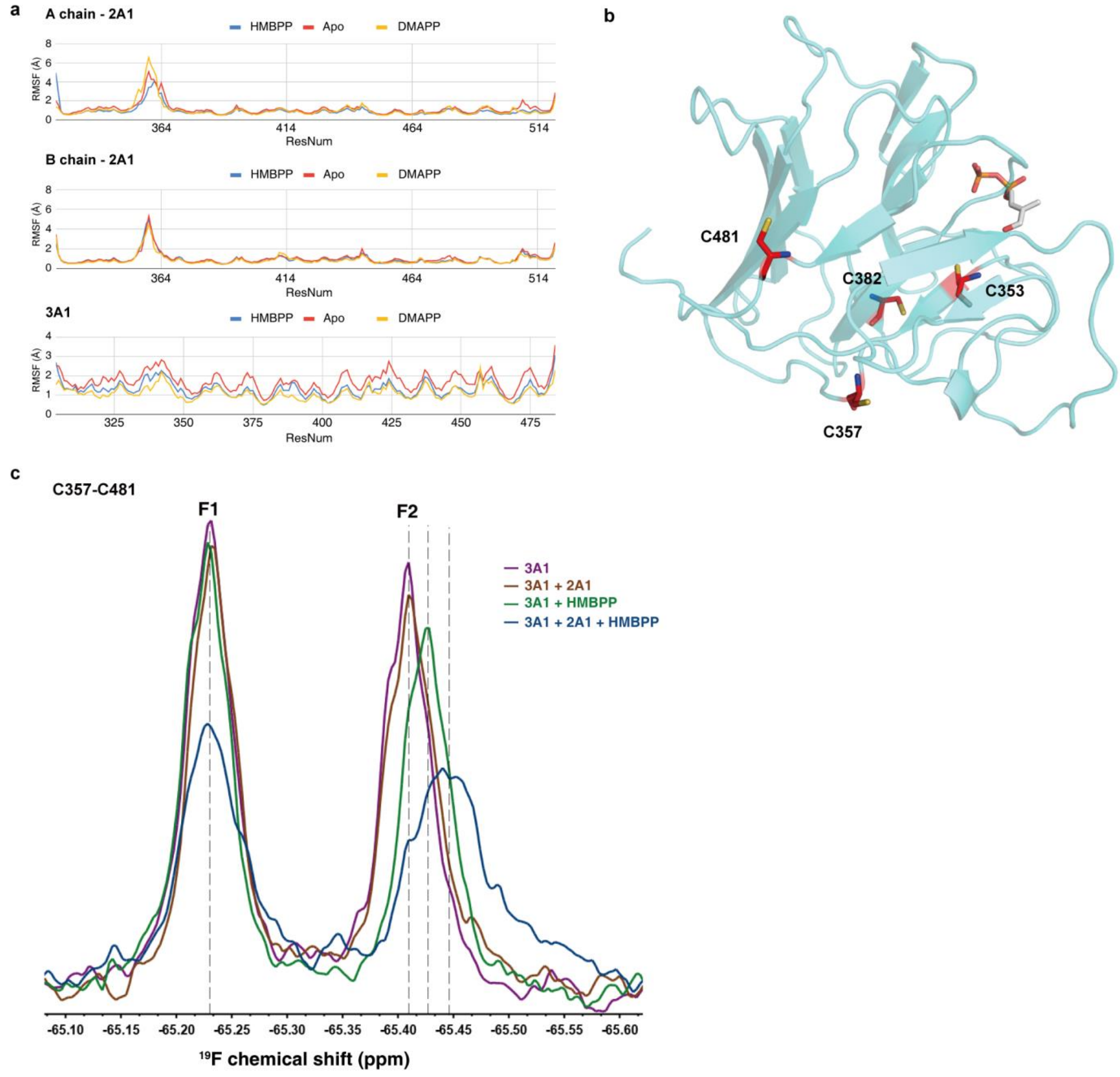

Supplementary Fig. 4| MD simulations and ^{19}F-NMR studies of 3A1 B30.2 fluctuations induced by pAg binding and upon association with 2A1 B30.2. a, Cα-RMSF profiles for simulations with the apo-complex as well as for HMBPP-bound and DMAPP-bound complexes. **b,** There are two deeply buried and non-reactive resides (C353 and C382) and two exposed cysteine residues (C357 and C481) in HMBPP-bound 3A1 B30.2 (in cyan, PDB: 5ZXK). **c,** ^{19}F-NMR spectra showing that upon addition of HMBPP and 2A1 30.2. to 3A1 B30.2, one TET signal F1 (-65.23 ppm) remained unchanged, while F2 (-65.41 ppm) changed.

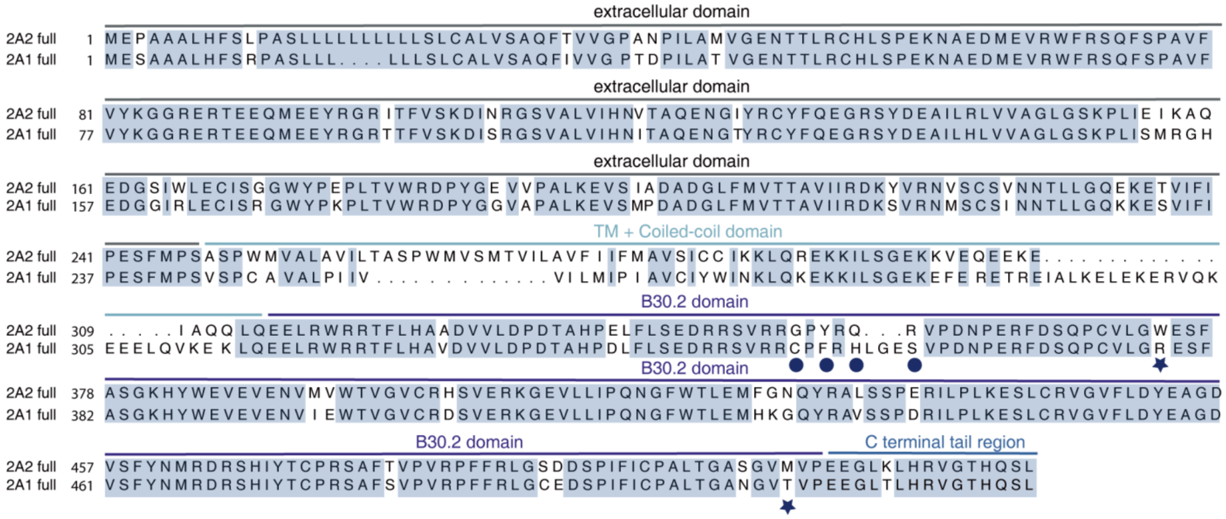

Supplementary Fig. 5| Sequence alignment of the full-length 2A1 and 2A2 proteins. BTN2A1 B30.2 and BTN2A2 B30.2 share 88.7% similarity. Blue stars indicate the residues in 2A2 (W374 and M506) analogous to 2A1 B30.2 residues (R378 and T510) identified as functionally relevant for intracellular association with 3A1. Note that 2A1 has an additional loop region (C353F355HLGES, shown by the blue circles) that is not present in the 2A2 structure (G352Y354QR). Based on these structural analyses, we generated the following 2A2 mutations: G352Y354QR, W374R, M506T, W374R/M506T, and G352Y354QR/W374R/M506T. But only two 2A2 B30.2 mutants (M506T and W374R/M506T) bind to the 3A1 B30.2 domain in the presence of HMBPP.

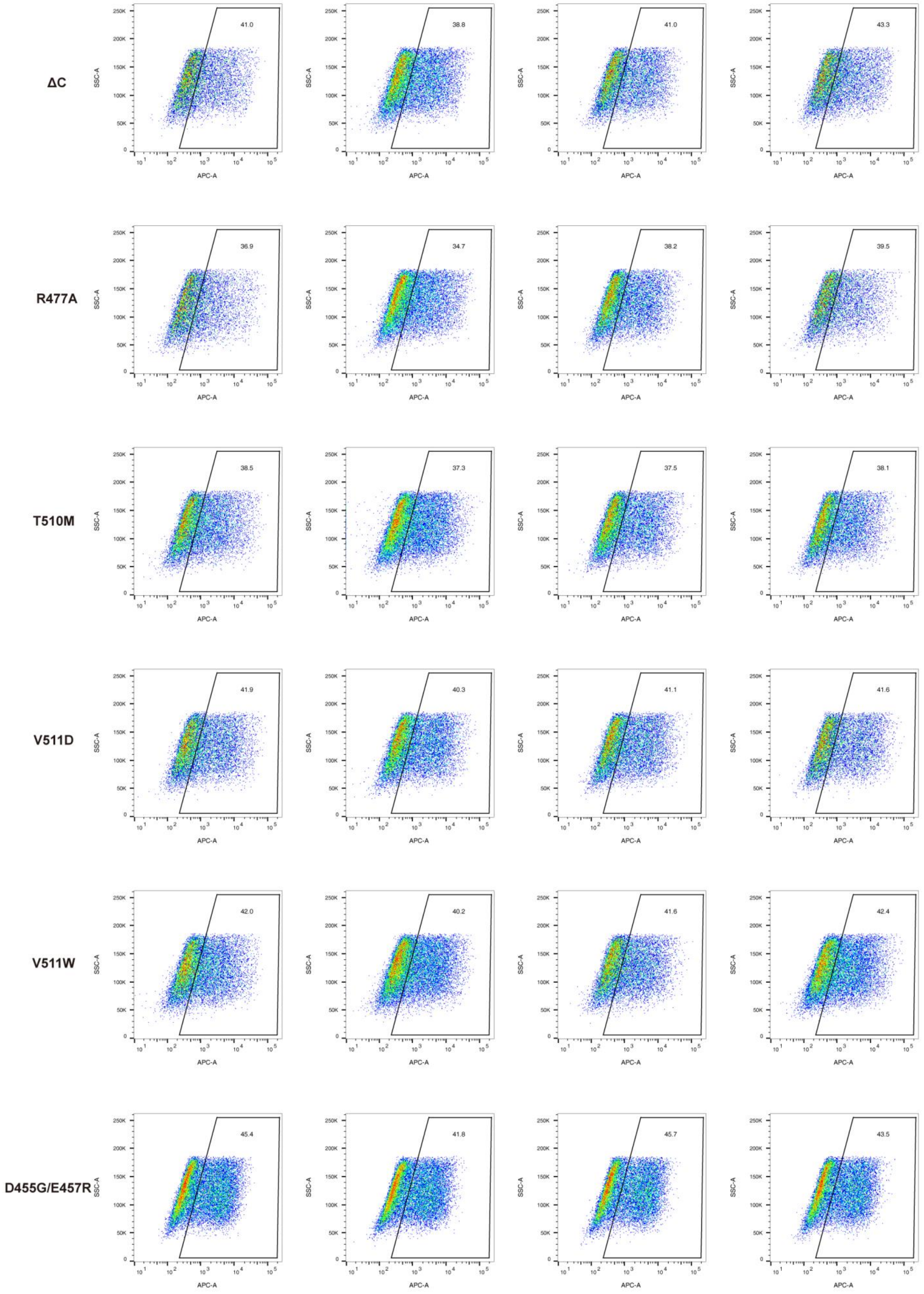
ΔC
41.0
38.8
41.0
43.3
R477A
36.9
34.7
38.2
39.5
T510M
38.5
37.3
37.5
38.1
V511D
41.9
40.3
41.1
41.6
V511W
42.0
40.2
41.6
42.4
D455G/E457R
45.4
41.8
45.7
43.5
SSC-A
APC-A

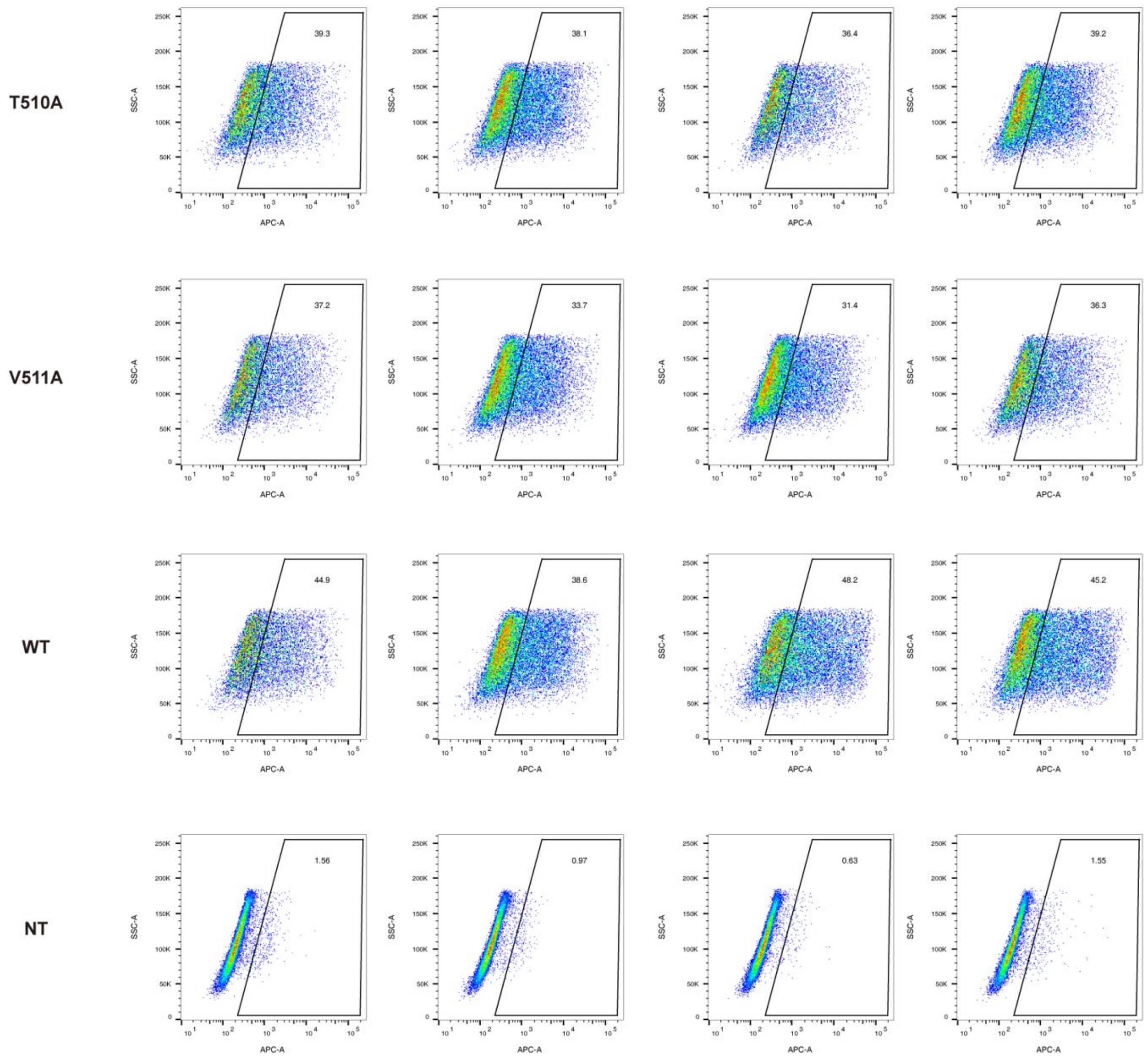

Supplementary Fig. 6| Gating strategy for flow cytometry analysis shown in Extended Data Fig. 2h. The percentage of His-tagged 2A1 mutant variants expression in BTN2A$^{-/-}$ 293T cells.

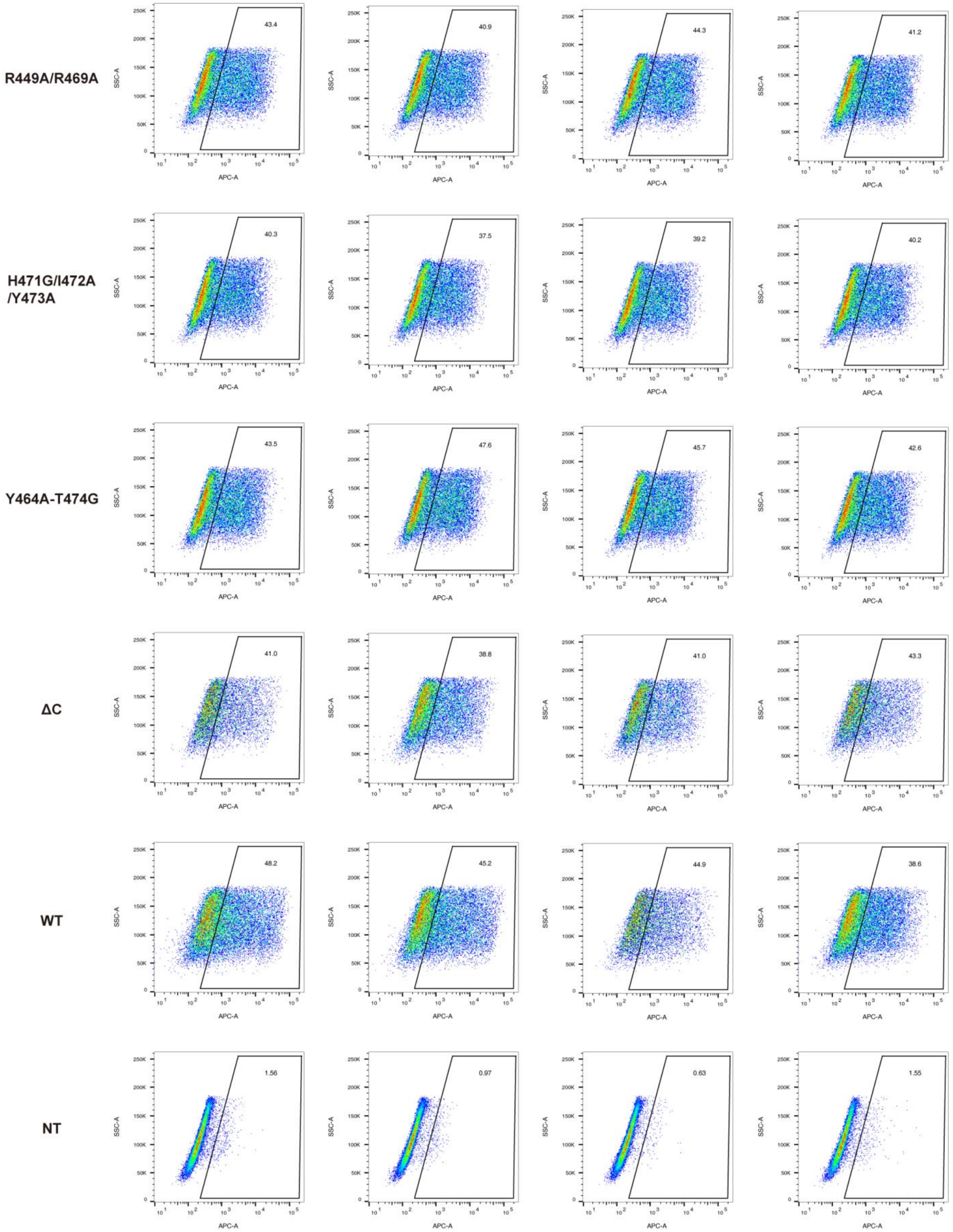

Supplementary Fig. 7| Gating strategy for flow cytometry analysis shown in Extended Data Fig. 3e. The percentage of His-tagged 2A1 mutant variants expression in BTN2A$^{-/-}$ 293T cells.

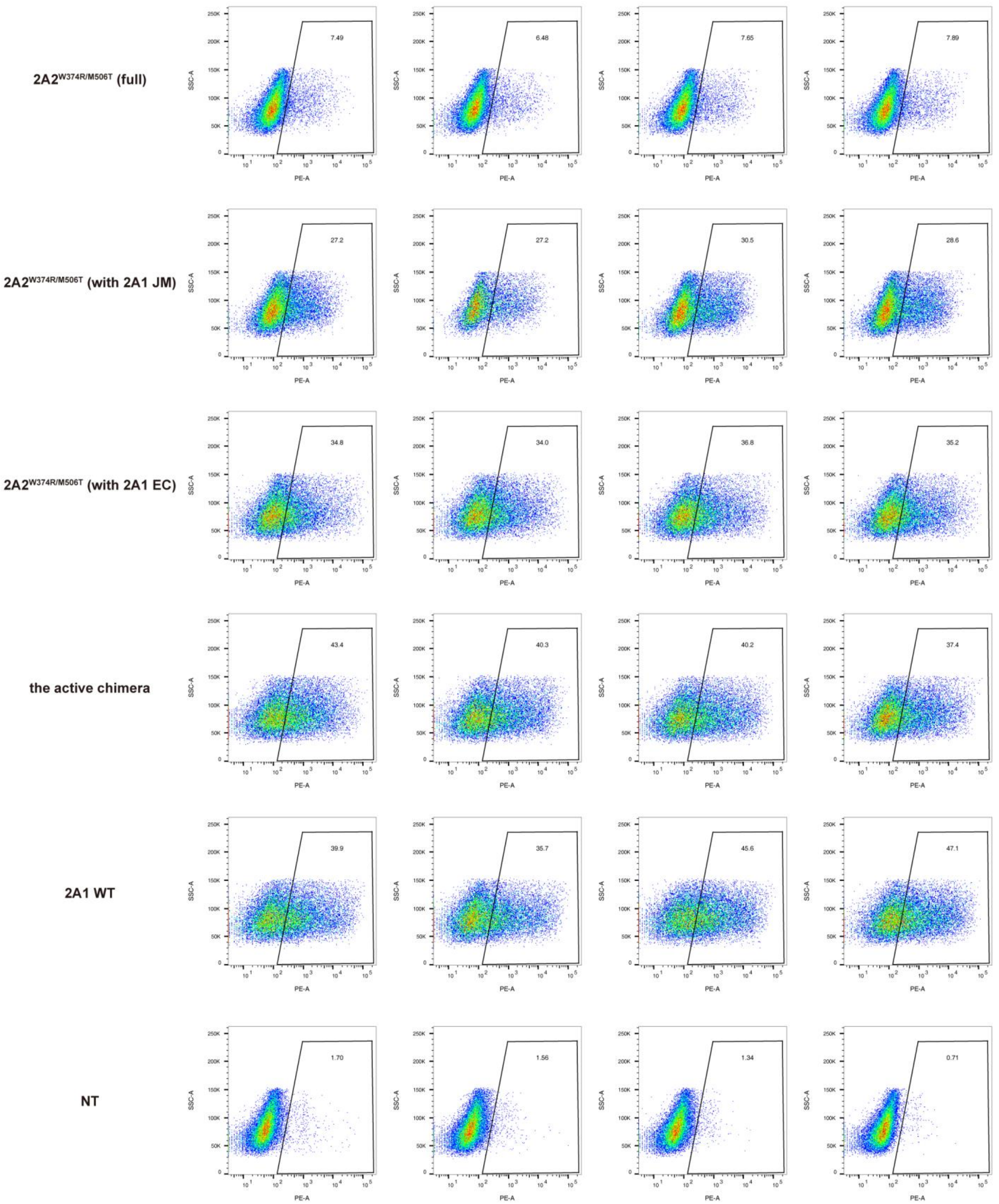

Supplementary Fig. 8| Gating strategy for flow cytometry analysis shown in Extended Data Fig. 6f. The percentage of His tagged chimeric variants of 2A2 (with the 2A1 JM, EC, or both JM and EC, as indicated).

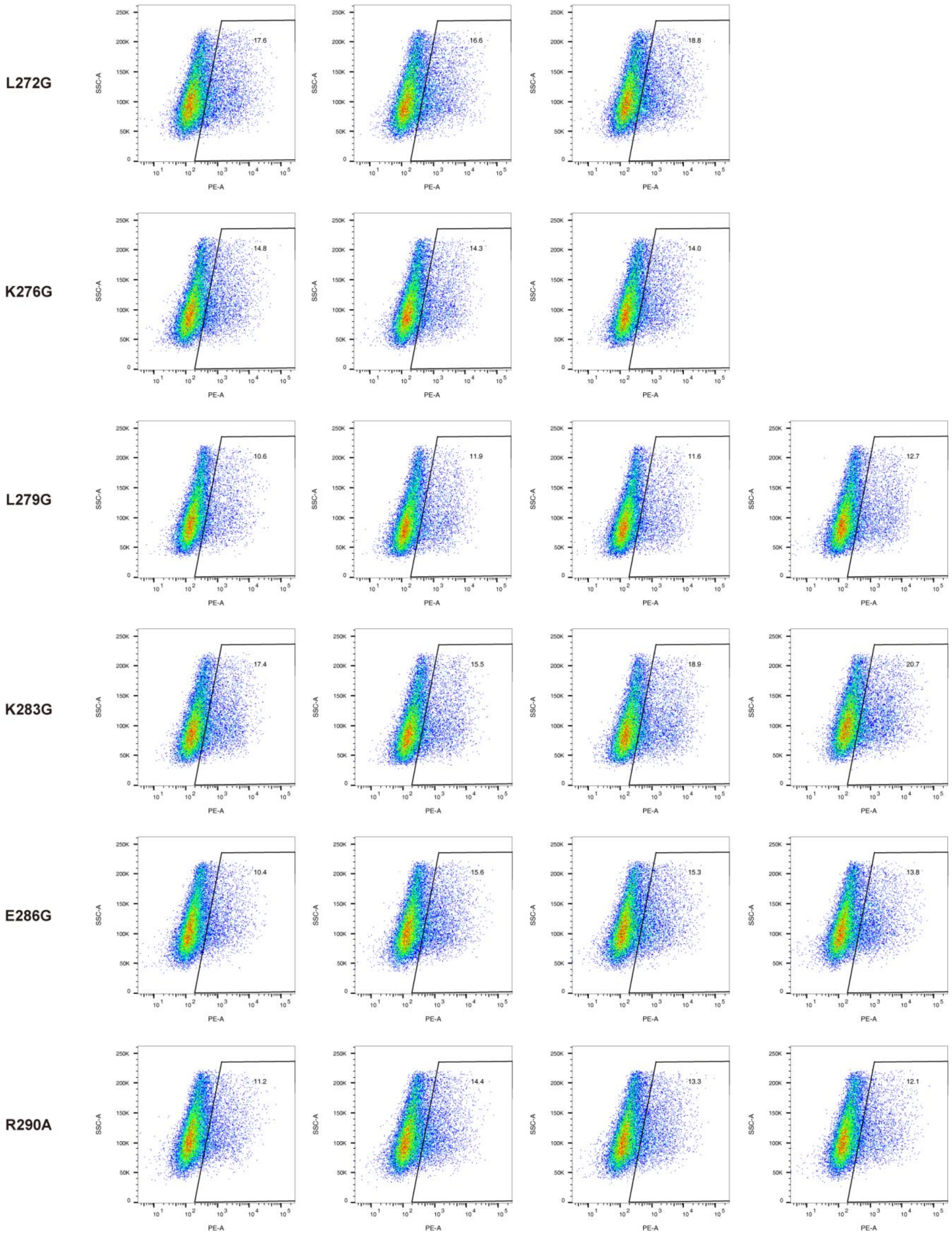
L272G
17.6
16.6
18.8
K276G
14.8
14.3
14.0
L279G
10.6
11.9
11.6
12.7
K283G
17.4
15.5
18.9
20.7
E286G
10.4
15.6
15.3
13.8
R290A
11.2
14.4
13.3
12.1
SSC-A
PE-A

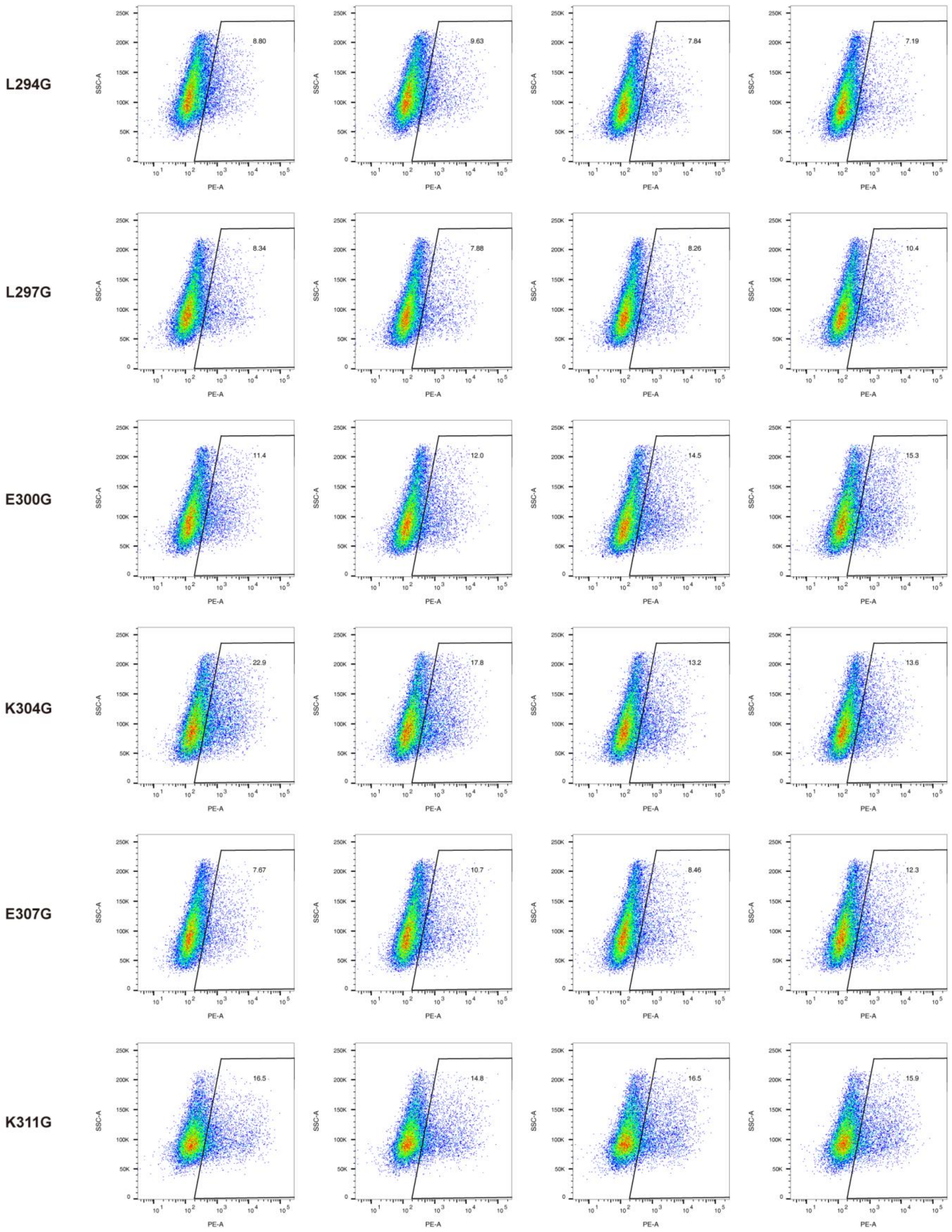
L294G
8.80
9.63
7.84
7.19
L297G
8.34
7.88
8.26
10.4
E300G
11.4
12.0
14.5
15.3
K304G
22.9
17.8
13.2
13.6
E307G
7.67
10.7
8.46
12.3
K311G
16.5
14.8
16.5
15.9
SSC-A
PE-A

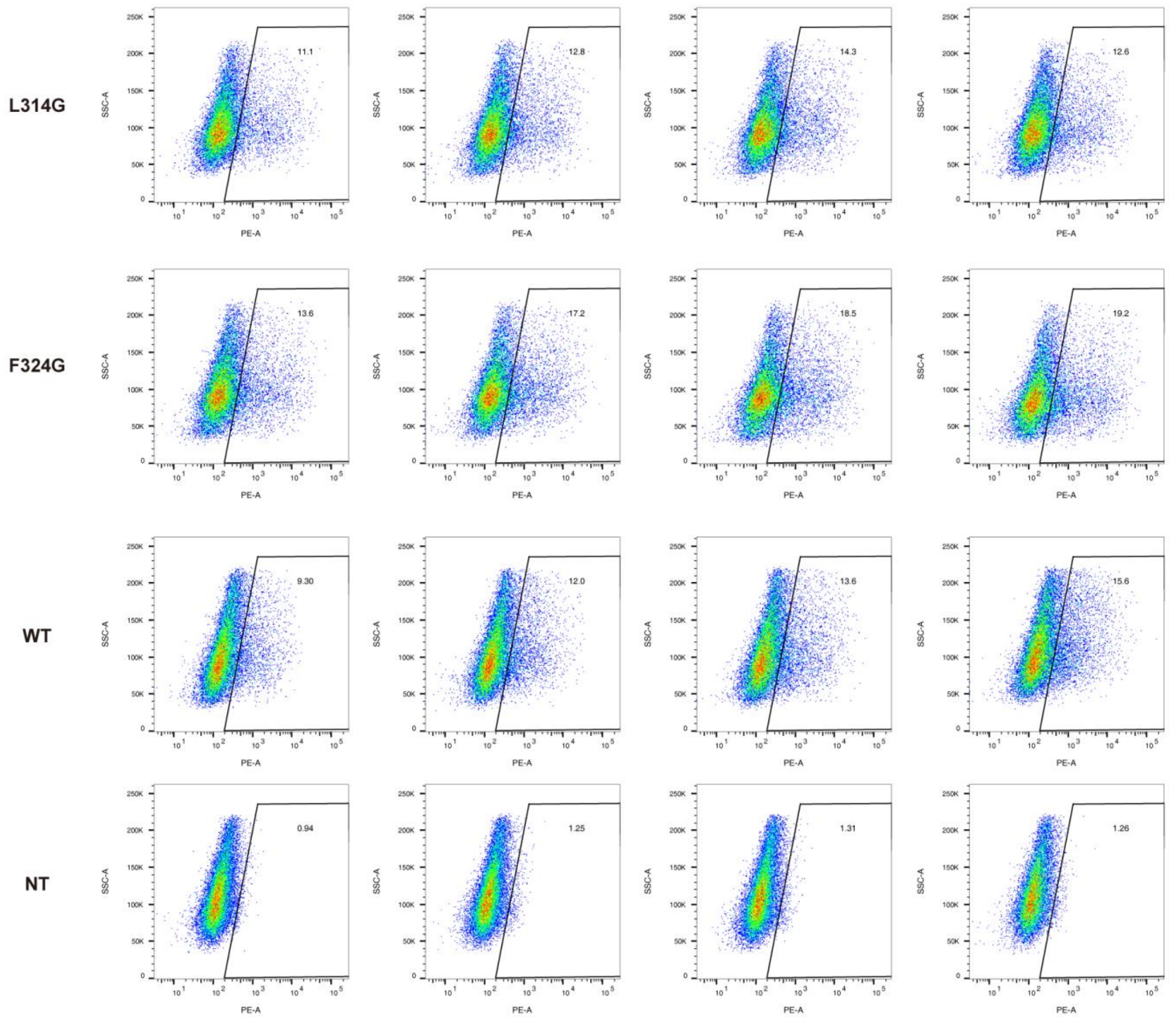

Supplementary Fig. 9| Gating strategy for flow cytometry analysis shown in Extended Data Fig. 7c. The percentage of His-tagged 2A1 JM mutant variants in BTN2A$^{-/-}$ 293T cells.

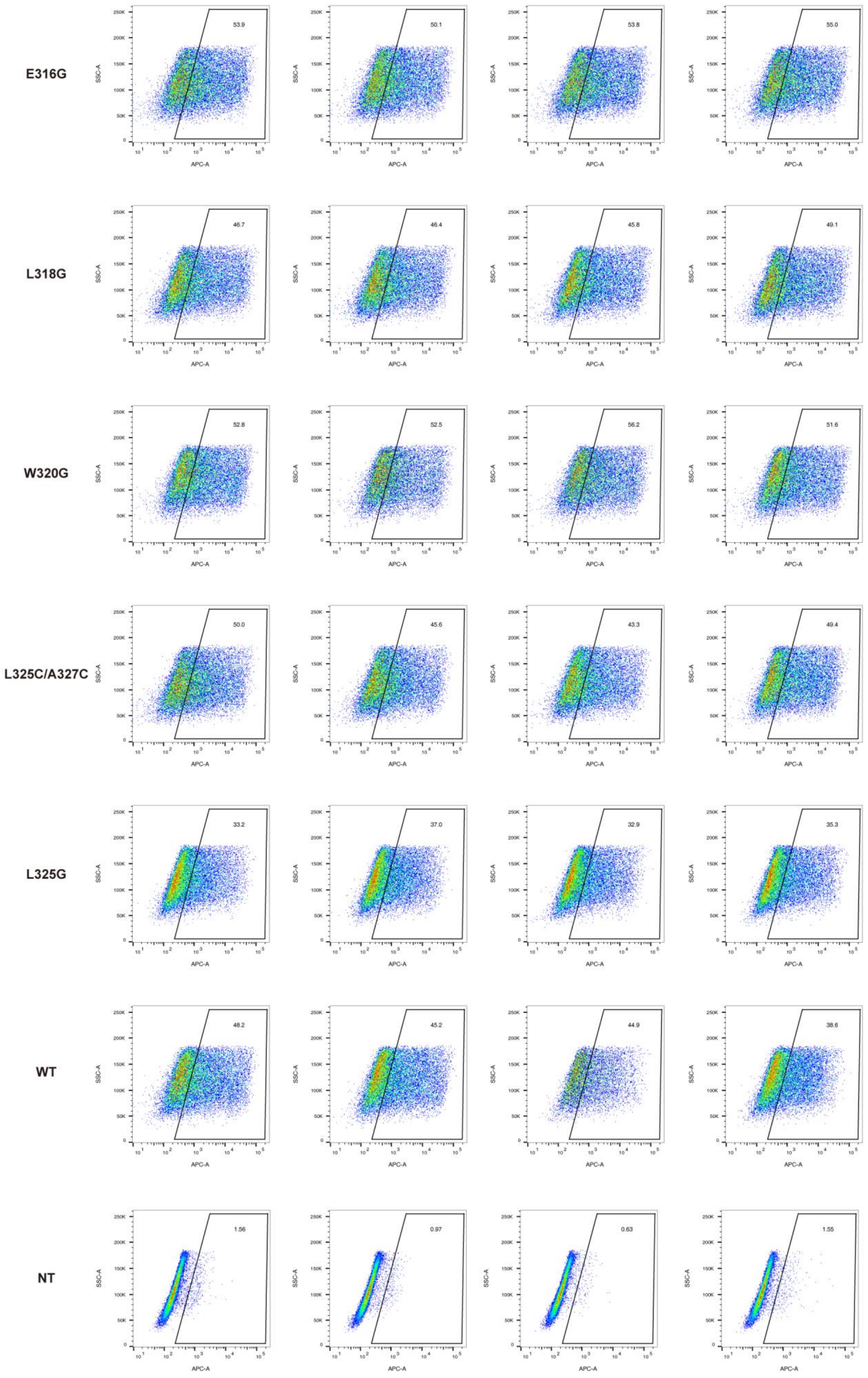

E316G
53.9
50.1
53.8
55.0
L318G
46.7
46.4
45.8
49.1
W320G
52.8
52.5
56.2
51.6
L325C/A327C
50.0
45.6
43.3
49.4
L325G
33.2
37.0
32.9
35.3
WT
48.2
45.2
44.9
38.6
NT
1.56
0.97
0.63
1.55
SSC-A
APC-A

Supplementary Fig. 10| Gating strategy for flow cytometry analysis shown in Extended Data Fig. 7d. The percentage of His-tagged 2A1 JM mutant variants in BTN2A$^{-/-}$ 293T cells.

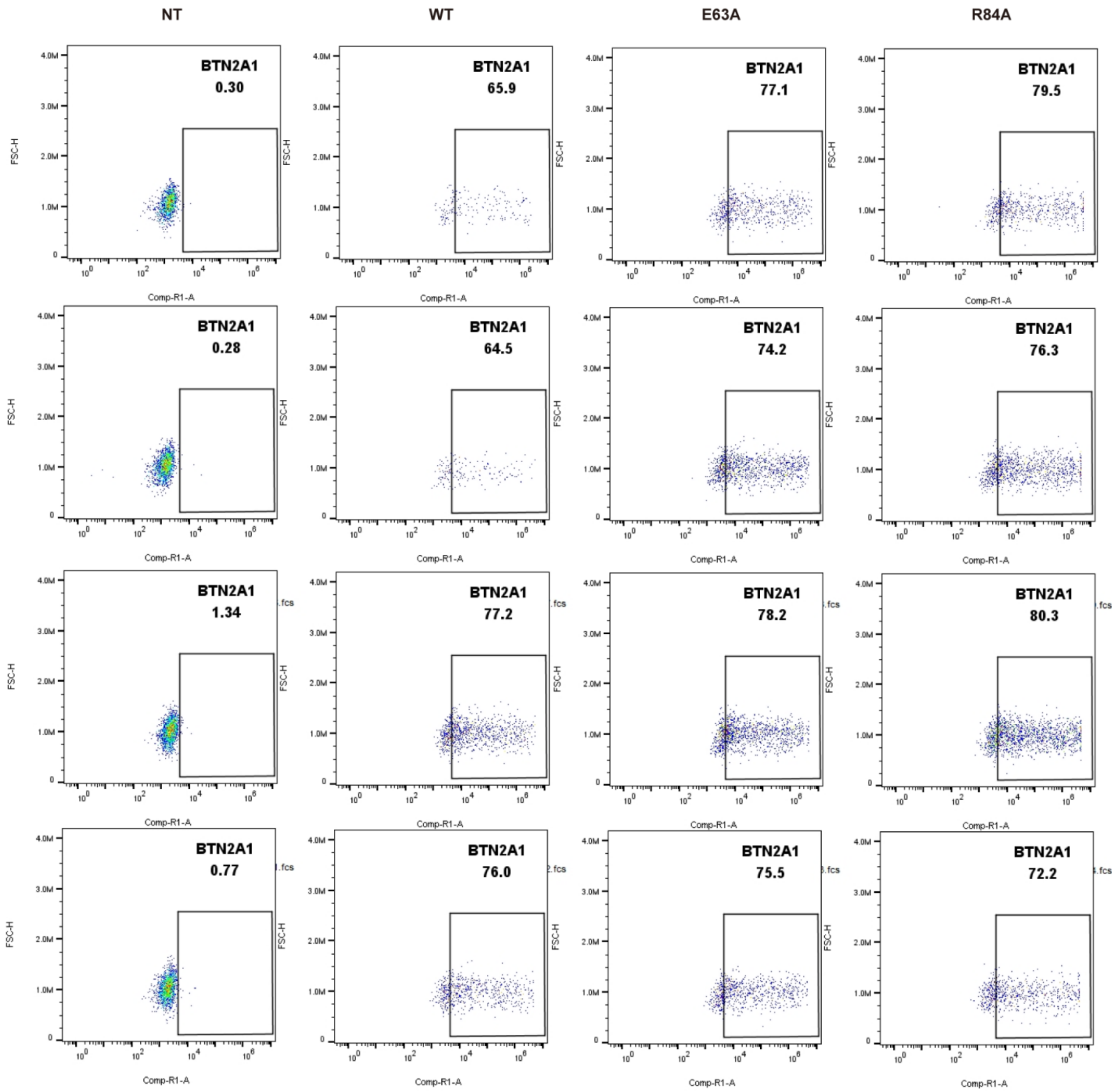

Supplementary Fig. 11| Gating strategy for flow cytometry analysis shown in Extended Data Fig. 7g. The percentage of His-tagged 2A1 EC mutant variants (NT, WT, E63A and R84A) expression in BTN2A$^{-/-}$ MIA PaCa-2 cells**.**

Supplementary Table 1| The BTN2A1-BTN3A1 interaction in the presence of HMBPP or DMAPP

PAg	BTN	Residue	Bond type
HMBPP	3A1 B30.2	H351, Y352, W391, M394, R412, R418, R469	H-bonding, salt bridge
	2A1 B30.2 A Chain	T510, V511	H-bonding
	2A1 B30.2 B Chain	R477	H-bonding, salt bridge
DMAPP	3A1 B30.2	H351, Y352, W391, M394, R412, R418, R469	H-bonding, salt bridge
	2A1 B30.2 A Chain	T510, V511	H-bonding
	2A1 B30.2 B Chain	R477	H-bonding, salt bridge

Supplementary Table 2| Interactions between 3A1 B30.2 and 2A1 B30.2

3A1 B30.2	2A1 B30.2 A Chain	2A1 B30.2 B Chain	Bond type
K336	E345, D346	-	H-bonding (water-mediated)
E337	R378	-	Salt bridge
K393	E513	-	Salt bridge
R418	E513	-	H-bonding
W350	-	D455	H-bonding (water-mediated)
W391	-	E457	H-bonding

Supplementary Table 3| Interactions between BTN2A1 A chain and B chain

2A1 A chain	2A1 B chain	Distance (Å)	Bond type
F324	H326	3.58	Pi-pi stacking
L325	A327	2.8	H-bonding
H326	F324	4.07	Pi-pi stacking
A327	L325	2.9	H-bonding
Y387	Y387	2.5	H-bonding
E389	R469	3.2	H-bonding
R439	E514	3.2	H-bonding
R449	M466, R467	2.6, 2.8	H-bonding
D460	A506	2.7	H-bonding
Y464	G505	2.7	H-bonding
M466	R449	2.7	H-bonding
R467	R449	3.0	H-bonding
R469	E389, P501	2.9, 3.0	Salt bridge, H-bonding
H471	H519, R520	3.0, 2.9	H-bonding
I472	L518	2.9	H-bonding
Y473	E514	2.6	H-bonding
T474	L516	3.0, 3.0	H-bonding
R477	A506, V509	2.9, 2.9	H-bonding
P501	R469	3.0	H-bonding
G505	Y464	2.7	H-bonding
A506	D460, R477	2.8, 2.8	H-bonding
V509	R477	2.7	H-bonding
E514	Y473, R439	2.7, 3.2	H-bonding
L516	T474	2.9, 2.9	H-bonding
L518	I472	2.8	H-bonding
H519	H471	3.0	H-bonding
R520	H471	2.9	H-bonding

Supplementary Table 4| Summary of cell activities and binding affinities of HMBPP analogs

Compounds	EC_{50} (M)[a]	K_D (M)[b]	CLogP[c]
**1**	3.875E-09	4.68E-08	-2.241
**2**	3.378E-05	3.45E-05	-0.454
**3**	4.934E-03	6.25E-04	-0.654
**4**	4.498E-07	3.26E-07	-1.712
**5**	1.302E-08	2.06E-07	-1.667
**6**	1.798E-06	1.69E-05	-1.932
**7**	1.461E-06	4.62E-05	-2.640
**8**	9.280E-07	1.25E-05	-0.741
**9**	3.499E-05	4.97E-05	-1.241
**10**	5.325E-06	1.15E-05	-1.041
**11**	8.710E-07	2.81E-05	-1.441

[a] The EC_{50} for MIA PaCa-2 cell lysis mediated by HMBPP analogs (Extended Data Fig. 5a) and Vγ9Vδ2 T cells.
[b] K_D for phosphoantigen binding to the pre-conditioned 2A1 B30.2 and 3A1 B30.2 as determined by isothermal titration calorimetry.
[c] The $\log_{10}$ of the oil/water partition coefficient P, computed by using ChemDraw.

Supplementary Table 5| List of primers used

Backbone	Primer name	Direction	Sequence	Vector
	B30.2 N-His	Forward	TGCCGCGCGGCAGCCATATGGAGAATCTTTATTTTCAGGGCGCTGGTGCTG GTGCTGAAGAATTGCGATGG	pET28a
		Reverse	CGGAGCTCGAATTCGGATCCTTATAGGCTCTGGTGGGT	
	B30.2 C-His	Forward	CTTTAAGAAGGAGATATACCATGGGAGAAGAATTGCGATGG	
		Reverse	CTCAGTGGTGGTGGTGGTGGTGCTCGAGTAGGCTCTGGTGGGTCCC	
	B30.2 ΔC	Forward	GAAGGAGATATACCATGGGAGAAGAATTG	
		Reverse	CTCAGTGGTGGTGGTGGTGGTGCTCGAGTGCAGGGCAGATGAAGATG	
BTN2A1	ΔC	Forward	GTACCGAGCTCGGATCCATGGAATCAGCTGCTGCCC	pET28a, pcDNA3.1
		Reverse	GGCCCTCTAGACTCGAGCGGCCGCCTACTTATCGTCGTCATCCTTGTAATC AGAGCCTCCACCCCCTGCAGGGCAGATGAAG	
	R477A	Forward	CATGTCCCGCCTCAGCCTTTTCCGTGCCTGTGAGGCCC	
		Reverse	AAGGCTGAGGCGGGACATGTGTAGATGTGCGATCTGTC	
	T510M	Forward	ATGGGGTCATGGTGCCTGAAGAGGGCCTGACACTTCAC	
		Reverse	TTCAGGCACCATGACCCCATTGGCTCCTGTGAGTGCAG	
	V511D	Forward	GGAGCCAATGGGGTCACGGATCCTGAAG	
		Reverse	TGTCAGGCCCTCTTCAGGATCCGTGACC	
	V511W	Forward	GGAGCCAATGGGGTCACGTGGCCTGAAG	
		Reverse	TGTCAGGCCCTCTTCAGGCCACGTGACC	
	T510A	Forward	CAATGGGGTCGCGGTGCCTGAAGAGGGCCTGACAC	
		Reverse	CTTCAGGCACCGCGACCCCATTGGCTCCTGTGAGT	
	V511A	Forward	TGGGGTCACGGCGCCTGAAGAGGGCCTGACACTTC	
		Reverse	CCTCTTCAGGCGCCGTGACCCCATTGGCTCCTGTG	
	L325C/A325C	Forward	AGAACATTCTGCCATTGTGTTGATGTGGTCCTGGATC	
		Reverse	CCACATCAACACAATGGCAGAATGTTCTTCTCCATCGC	
	R449A/R469A	Forward	GTCCCTTTGCGCGGTGGGCGTCTTCCTGGACTA CATGAGGGACGCATCGCACATCTACACATGTCCCC	
		Reverse	AGACGCCCACCGCGCAAAGGGACTCCTTCAAAG GTAGATGTGCGATGCGTCCCTCATGTTGTAGAAGG	
	H471G/I472A/Y473A	Forward	GGGACAGATCGGGCGCCGCCACATGTCCCCGTTCAGCC	
		Reverse	GGGACATGTGGCGGCGCCCGATCTGTCCCTCATGTTGT	
	Y464A-T474G	Forward	CTGGAGATGTCTCCTTCGCCGCTGGCGCCGCTGGCGCCGCTGGCGCTGGCT GTCCCCGTTCAGCCTTTTCCGTG	
		Reverse	CTGAACGGGGACAGCCAGCGCCAGCGGCGCCAGCGGCGCCAGCGGCGAA GGAGACATCTCCAGCTTCATAGTC	
	L272G	Forward	GATCAACAAAGGCCAAAAGGAAAAAAAGATTCTG	
		Reverse	CTTTTGGCCTTTGTTGATCCAATAGATGCATACG	
	K276G	Forward	AAAAGGAAGGAAAGATTCTGTCAGGGGAAAAGG	
		Reverse	GAATCTTTCCTTCCTTTTGGAGTTTGTTGATC	
	L279G	Forward	AAAAGATTGGGTCAGGGGAAAAGGAGTTTGAA	
		Reverse	TTCCCCTGACCCAATCTTTTTTTCCTTTTGGA	
	K283G	Forward	CAGGGGAAGGGGAGTTTGAACGGGAAACAAG	
		Reverse	CAAACTCCCCTTCCCCTGACAGAATCTTTTT	
	E286G	Forward	AGGAGTTTGGACGGGAAACAAGAGAAATTGCT	
		Reverse	GTTTCCCGTCCAAACTCCTTTTCCCCTGACAG	
	R290A	Forward	GGGAAACAGGAGAAATTGCTCTAAAGGAACTG	
		Reverse	GCAATTTCTCCTGTTTCCCGTTCAAACTCCTTT	
	L294G	Forward	AAATTGCTGGAAAGGAACTGGAGAAAGAACGTG	
		Reverse	AGTTCCTTTCCAGCAATTTCTCTTGTTTCCCG	
	L297G	Forward	TAAAGGAAGGGGAGAAAGAACGTGTGCAAAAA	
		Reverse	CTTTCTCCCCTTCCTTTAGAGCAATTTCTCTTG	
	E300G	Forward	GGAGAAAGGACGTGTGCAAAAAGAGGAAGAAC	
		Reverse	TGCACACGTCCTTTCTCCAGTTCCTTTAGAGC	
	K304G	Forward	GTGTGCAAGGAGAGGAAGAACTTCAAGTAAAA	
		Reverse	TCTTCCTCTCCTTGCACACGTTCTTTCTCCAG	
	E307G	Forward	AAGAGGAAGGACTTCAAGTAAAAGAGAAACTT	
		Reverse	ACTTGAAGTCCTTCCTCTTTTTGCACACGTTC	
	K311G	Forward	TTCAAGTAGGAGAGAAACTTCAAGAAGAATTG	
		Reverse	AGTTTCTCTCCTACTTGAAGTTCTTCCTCTTT	
	L314G	Forward	AAGAGAAAGGGCAAGAAGAATTGCGATGGAGA	
		Reverse	TCTTCTTGCCCTTTCTCTTTTACTTGAAGTT	
	E316G	Forward	AAACTTCAAGGAGAATTGCGATGGAGAAGAACAT	
		Reverse	CATCGCAATTCTCCTTGAAGTTTCTCTTTTACTTG	
	L318G	Forward	TCAAGAAGAAGGGCGATGGAGAAGAACATTCTTACATG	

		Reverse	TCTCCATCGCCCTTCTTCTTGAAGTTTCTCTTTT	
	W320A	Forward	AAGAATTGCGAGCGAGAAGAACATTCTTACATGC	
		Reverse	ATGTTCTTCTCGCTCGCAATTCTTCTTGAAGTTTCTC	
	F324G	Forward	GAAGAACAGGCTTACATGCTGTTGATGTGGTC	
		Reverse	GCATGTAAGCCTGTTCTTCTCCATCGCAATTC	
	L325G	Forward	AAGAACATTCGGACATGCTGTTGATGTGGTCCTG	
		Reverse	ATCAACAGCATGTCCGAATGTTCTTCTCCATCGCA	
	D455G/E457R	Forward	CGGGTGGGCGTCTTCCTGGGATATCGGGCTGGAG	
		Reverse	GAAGGAGACATCTCCAGCCCGATATCCCAGGAAG	
	E63A	Forward	TGAGGACATGGCGGTGCGGTGGTTCCGGTCT	
		Reverse	ACCACCGCACCGCCATGTCCTCAGCATTTTTCT	
	R84A	Forward	AGGTGGCAGAGAGGCAACAGAGGAGCAGATGGAGGA	
		Reverse	CTCCATCTGCGCCTCTGTTCTCTCTCTGCCACCT	
BTN2A2	B30.2 N-His	Forward	TGCCGCGCGGCAGCCATATGGAGAATCTTTATTTTCAGGGCGCTGGTGCTG GTGCTGAAGAATTGCGATGG	pET28a
		Reverse	CGGAGCTCGAATTCGGATCCCTATAGGCTCTGGTG	
	G352Y354QR	Forward	TGCCCCTTCAGGCACCTAGGGGAGAGCGTGCCTGACAACCCAGAGAGATT	pET28a, pcDNA3.1
		Reverse	TGCCCCTTCAGGCACCTAGGGGAGAGCGTGCCTGACAACCCAGAGAGATT	
	W374R	Forward	TCCTGGGACGGGAGAGCTTCGCCTCAGGGAAACATTAC	
		Reverse	AAGCTCTCCCGTCCCAGGACACAAGGCTGACTGTCGAA	
	M506T	Forward	GTGGGGTCACGGTGCCTGAAGAGGGCCTGAAACTTCAC	
		Reverse	TCAGGCACCGTGACCCCACTGGCTCCTGTGAGTGCAGG	
	W374R/M506T	Forward	TCCTGGGACGGGAGAGCTTCGCCTCAGGGAAACATTACGTGGGGTCACGG TGCCTGAAGAGGGCCTGAAACTTCAC	
		Reverse	AAGCTCTCCCGTCCCAGGACACAAGGCTGACTGTCGAATCAGGCACCGTG ACCCCACTGGCTCCTGTGAGTGCAGG	
CRISPR Cas9	BTN2A1 sgRNA	Forward	CACCGGCCATCCTGCACCTCGTAG	PX458-pSpCas9(BB)-2A-GFP-MCS
		Reverse	AAACCTACGAGGTGCAGGATGGCC	
	BTN2A2 sgRNA1	Forward	CACCGTGCCACCACGAGGCGTAGGA	
		Reverse	AAACTCCTACGCCTCGTGGTGGCA	
	BTN2A2 sgRNA2	Forward	CACCGCCATCCAGGGAGATGCGGTC	
		Reverse	AAACGACCGCATCTCCCTGGATGG	
