# Supplementary material for: Phosphoantigens glue butyrophilin 3A1 and 2A1 to activate Vγ9Vδ2 T cells
Source: Nature. 2023 Sep 6;621(7980):840–8. doi: 10.1038/s41586-023-06525-3 (PMC10533412; doi:10.1038/s41586-023-06525-3)
Supplement: Supplementary file 2 — Reporting Summary [file 41586_2023_6525_MOESM2_ESM.pdf]

nature portfolio

Corresponding author(s): Yonghui Zhang, Rey-Ting Guo

Last updated by author(s): Jul 30, 2023

Reporting Summary

Nature Portfolio wishes to improve the reproducibility of the work that we publish. This form provides structure for consistency and transparency in reporting. For further information on Nature Portfolio policies, see our Editorial Policies and the Editorial Policy Checklist.

Statistics

For all statistical analyses, confirm that the following items are present in the figure legend, table legend, main text, or Methods section.

n/a	Confirmed	
☐	☒	The exact sample size (n) for each experimental group/condition, given as a discrete number and unit of measurement
☐	☒	A statement on whether measurements were taken from distinct samples or whether the same sample was measured repeatedly
☐	☒	The statistical test(s) used AND whether they are one- or two-sided<br>*Only common tests should be described solely by name; describe more complex techniques in the Methods section.*
☒	☐	A description of all covariates tested
☐	☒	A description of any assumptions or corrections, such as tests of normality and adjustment for multiple comparisons
☐	☒	A full description of the statistical parameters including central tendency (e.g. means) or other basic estimates (e.g. regression coefficient) AND variation (e.g. standard deviation) or associated estimates of uncertainty (e.g. confidence intervals)
☐	☒	For null hypothesis testing, the test statistic (e.g. F, t, r) with confidence intervals, effect sizes, degrees of freedom and P value noted<br>*Give P values as exact values whenever suitable.*
☒	☐	For Bayesian analysis, information on the choice of priors and Markov chain Monte Carlo settings
☒	☐	For hierarchical and complex designs, identification of the appropriate level for tests and full reporting of outcomes
☒	☐	Estimates of effect sizes (e.g. Cohen's d, Pearson's r), indicating how they were calculated

Our web collection on statistics for biologists contains articles on many of the points above.

Software and code

Policy information about availability of computer code

Data collection	The data of apo-BTN2A1 B30.2 domain and BTN2A1-BTN3A1 B30.2 with HMBPP or DMAPP were collected at the Shanghai Synchrotron Radiation Facility (SSRF), beamlines BL17U1, BL18U1, and BL19U1, and data were processed using the HKL-2000 program.<br>X-ray diffraction data of BTN2A2 B30.2W374R/M506T, apo-VpBTN3 B30.2 ΔC, and the complex crystals of VpBTN3 B30.2 ΔC with HMBPP/DMAPP/IPP, VpBTN2-VpBTN3 B30.2 with HMBPP and BTN3A1 B30.2 domain with compound 8 were obtained at the in-house beamline BRUKER D8 VENTURE at Hubei University and datasets were processed with PROTEUM3 (Bruker AXS GmbH).<br>The data of BTN3A1 B30.2 domain with compound 4 and 5 were collected at the beamline TPS05A of the National Synchrotron Radiation Research Center (NSRRC, Hsinchu, Taiwan) and processed by using the HKL-2000 program.
Data analysis	COOT 0.9.8.1 and REFMAC, GraphPad Prism 9.0 software (GraphPad, La Jolla, CA, USA), FlowJo 10.6.2, JPK image processing software 6.0, MicroCal PEAQ-ITC anaysis software 1.1.0.1262, PyMOL 2.5.2, Chemdraw20.0, Schrödinger Release 2021-1/2/3, Schrödinger Release 2022-2, Schrödinger Release 2023-1, PIPER (Schrödinger Release 2023-1)

For manuscripts utilizing custom algorithms or software that are central to the research but not yet described in published literature, software must be made available to editors and reviewers. We strongly encourage code deposition in a community repository (e.g. GitHub). See the Nature Portfolio guidelines for submitting code & software for further information.

Data

Policy information about availability of data

All manuscripts must include a data availability statement. This statement should provide the following information, where applicable:

- Accession codes, unique identifiers, or web links for publicly available datasets
- A description of any restrictions on data availability
- For clinical datasets or third party data, please ensure that the statement adheres to our policy

Any additional information required to reanalyze the data available in a publicly accessible repository. The crystal structures are deposited in the Protein Data Bank under PDB: 8IGT, 8JYE, 8JYC, 8IH4, 8JYB, 8JY9, 8JYF, 8JYA, 8HJT, 8IZE, 8IZG and 8IXV. The structure data used from the Protein Data Bank are listed below: 5ZXK, 4F80, 1HXM, 4V1P and 6J06.

Research involving human participants, their data, or biological material

Policy information about studies with human participants or human data. See also policy information about sex, gender (identity/presentation), and sexual orientation and race, ethnicity and racism.

Reporting on sex and gender	N/A
Reporting on race, ethnicity, or other socially relevant groupings	N/A
Population characteristics	N/A
Recruitment	N/A
Ethics oversight	N/A

Note that full information on the approval of the study protocol must also be provided in the manuscript.

Field-specific reporting

Please select the one below that is the best fit for your research. If you are not sure, read the appropriate sections before making your selection.

☒ Life sciences ☐ Behavioural & social sciences ☐ Ecological, evolutionary & environmental sciences

For a reference copy of the document with all sections, see nature.com/documents/nr-reporting-summary-flat.pdf

Life sciences study design

All studies must disclose on these points even when the disclosure is negative.

Sample size	The sample size and the results of statistical analyses are described in Figure legends.
Data exclusions	Some data points were not successful and excluded from the assays. They were indicated as NaN in Source Data.
Replication	Experiment was repeated at least twice and the results were successfully reproduced. The number of biological and technical replicates were indicated in the figure legends.
Randomization	Data variability was controlled by multiple biological replicate and multiple technical replicates.
Blinding	For all experiments, there are both negative and positive controls and all the results were obtained in parallel using the same setting, and each treatment was assigned to a number during the experiment.

Reporting for specific materials, systems and methods

We require information from authors about some types of materials, experimental systems and methods used in many studies. Here, indicate whether each material, system or method listed is relevant to your study. If you are not sure if a list item applies to your research, read the appropriate section before selecting a response.

Materials & experimental systems

n/a	Involved in the study
☐	☒ Antibodies
☐	☒ Eukaryotic cell lines
☒	☐ Palaeontology and archaeology
☒	☐ Animals and other organisms
☒	☐ Clinical data
☒	☐ Dual use research of concern
☒	☐ Plants

Methods

n/a	Involved in the study
☒	☐ ChIP-seq
☐	☒ Flow cytometry
☒	☐ MRI-based neuroimaging

Antibodies

Antibodies used	APC anti-HIS antibody: Biolegend, Cat#362605, 1:100<br>PE anti-HIS antibody: Biolegend, Cat#362603, 1:100
Validation	All antibodies were validated to determined their optimal concentration.

Eukaryotic cell lines

Policy information about cell lines and Sex and Gender in Research

Cell line source(s)	MIA PaCa-2 cells (ATCC), HEK293T cells (ATCC), CHO-K1 cells (ATCC).
Authentication	Cell lines purchased from ATCC were not further authenticated.
Mycoplasma contamination	None of the cell lines used in this study tested positive for mycoplasma.
Commonly misidentified lines (See ICLAC register)	No commonly misidentified lines were used in this study.

Flow Cytometry

Plots

Confirm that:

☒ The axis labels state the marker and fluorochrome used (e.g. CD4-FITC).

☒ The axis scales are clearly visible. Include numbers along axes only for bottom left plot of group (a 'group' is an analysis of identical markers).

☒ All plots are contour plots with outliers or pseudocolor plots.

☒ A numerical value for number of cells or percentage (with statistics) is provided.

Methodology

Sample preparation	BTN2A-/- 293T (2A1/2A2 KO) cells were transfected with WT or mutant plasmids with an N terminal 6 × His tag, and then cells were stained with APC/PE anti-his antibody (Biolegend) for 30 min at 4 degree celsius.<br>BTN2A-/- MIA PaCa-2 (2A1/2A2 KO) cells were infected with lentivirus bearing BTN2A1 WT or ΔC mutant transgene with an N terminal 6 × His tag. Cells were stained with APC/PE anti-his antibody for 30 min at 4 degree celsius, and then sorted APC/PE positive cells using Moflo Astrios EQ (Beckman Coulter).
Instrument	Beckman Moflo AstriosEQ, LSRFortessa, BD FACS AriaII
Software	Analyzed with FlowJo 10.6.2 software.
Cell population abundance	We detected the BTN2A1 expression level at the plasma membrane by APC/PE positive cell populations.
Gating strategy	The FSC/SSC gates were applied. The cells were separated into APC/PE positive and negative cell populations.

☒ Tick this box to confirm that a figure exemplifying the gating strategy is provided in the Supplementary Information.
